# A flat embedding method for transmission electron microscopy reveals an unknown mechanism of tetracycline

**DOI:** 10.1038/s42003-021-01809-8

**Published:** 2021-03-08

**Authors:** Michaela Wenzel, Marien P. Dekker, Biwen Wang, Maroeska J. Burggraaf, Wilbert Bitter, Jan R. T. van Weering, Leendert W. Hamoen

**Affiliations:** 1 Bacterial Cell Biology, Swammerdam Institute for Life Sciences, University of Amsterdam, 1098 XH Amsterdam, The Netherlands; 2 Department of Medical Microbiology and Infection Control, Amsterdam University Medical Centers - Location VUMC, 1081 HZ Amsterdam, The Netherlands; 3 Chemical Biology, Department for Biology and Biological Engineering, Chalmers University of Technology, 412 96 Gothenburg, Sweden; 4 Department of Clinical Genetics, Center for Neurogenomics and Cognitive Research (CNCR), Neuroscience Campus Amsterdam, Amsterdam University Medical Centers - Location VUMC, 1081 HZ Amsterdam, The Netherlands; 5 Department of Molecular Cell Biology, Amsterdam Institute for Molecules, Medicines, and Systems, Faculty of Science, Vrije Universiteit Amsterdam, 1081 HZ Amsterdam, The Netherlands

**Keywords:** Cell biology, Microbiology

## Abstract

Transmission electron microscopy of cell sample sections is a popular technique in microbiology. Currently, ultrathin sectioning is done on resin-embedded cell pellets, which consumes milli- to deciliters of culture and results in sections of randomly orientated cells. This is problematic for rod-shaped bacteria and often precludes large-scale quantification of morphological phenotypes due to the lack of sufficient numbers of longitudinally cut cells. Here we report a flat embedding method that enables observation of thousands of longitudinally cut cells per single section and only requires microliter culture volumes. We successfully applied this technique to *Bacillus subtilis*, *Escherichia coli, Mycobacterium bovis*, and *Acholeplasma laidlawii*. To assess the potential of the technique to quantify morphological phenotypes, we monitored antibiotic-induced changes in *B. subtilis* cells. Surprisingly, we found that the ribosome inhibitor tetracycline causes membrane deformations. Further investigations showed that tetracycline disturbs membrane organization and localization of the peripheral membrane proteins MinD, MinC, and MreB. These observations are not the result of ribosome inhibition but constitute a secondary antibacterial activity of tetracycline that so far has defied discovery.

## Abstract (web summary)

Wenzel et al. developed a flat embedding method for TEM, which increases the fraction of appropriately orientated cells per image, making it easier to quantify cell morphology. They find that tetracycline-treated *B. subtilis* shows membrane lesions and that tetracycline action is via direct perturbation of bacterial cell membrane in addition to its known ribosome inhibition activity.

## Introduction

Transmission electron microscopy (TEM) is a powerful tool to examine the morphology and ultrastructure of bacterial cells. There are many bacterial embedding protocols for TEM^1–5^, but the basic procedure, i.e., embedding of cell pellets as small nuggets into resin blocks, has not changed since the beginning of electron microscopy research on bacteria 60 years ago^4,6,7^. This technique has two major shortcomings. Most importantly, it results in random orientations of cells in the ultrathin sections. This is a critical limitation when examining rod-shaped and other non-coccoid bacterial species, since the vast majority of cells are randomly cross-sectioned, and the number of complete longitudinally cut cells is generally so low that robust quantification and population-wide studies are not feasible. Another limitation is that acquiring a concentrated cell pellet often requires relatively large culture volumes typically in the range of 10 to 50 ml^6,8,9^. This can be problematic when studying the mode of action of experimental antimicrobial compounds, whose synthesis or purification is laborious and expensive.

We have addressed these problems by developing an embedding technique that enables observation of a large number of cells oriented in one plane by immobilizing bacterial samples on a flat surface of either agarose or glass. This relatively simple method does not require any expensive equipment and can be adapted for any microorganism. We have successfully used this method with the Gram-positive bacterium *Bacillus subtilis*, the Gram-negative bacterium *Escherichia coli*, the tuberculosis vaccine strain *Mycobacterium bovis* Bacillus Calmette–Guérin (BCG), and the cell wall-less mycoplasma species *Acholeplasma laidlawii*. This flat embedding technique allowed the quantification of morphological changes in bacteria treated with different antibiotics. This led to the surprising discovery that the well-known ribosome inhibitor tetracycline does not only block translation but also directly disturbs the bacterial cell membrane. This additional mechanism of action has remained hidden for over 50 years despite the fact that tetracyclines are one of the most commonly used antibiotic groups in both human and veterinary medicine^10^.

## Results

### Alignment of cells on agarose

**Fig. 1:**
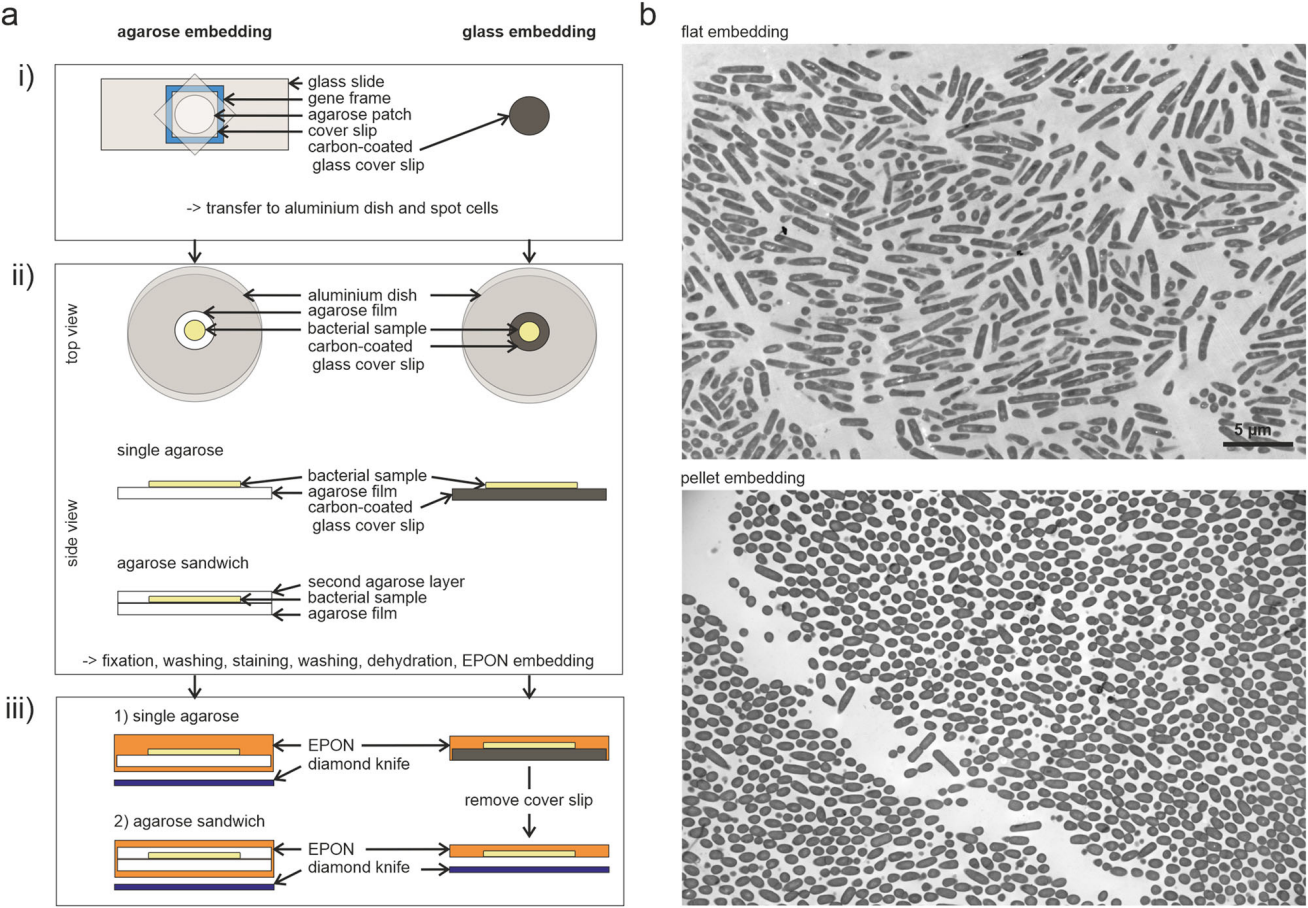
The flat embedding technique. **a** Schematic representation of the flat embedding workflow including embedding on a single layer of agarose, embedding in an agarose sandwich (used here for mycobacteria), and embedding on carbon-coated glass coverslips. (i) Preparation of the surface. Uniform thickness of the agarose film is ensured by using a gene frame as spacer. (ii) The agarose or glass surface is transferred to an aluminum dish and a small drop of cell sample is spotted on top of the surface and allowed to evaporate under a slight air flow. For the agarose sandwich approach, a second flat layer of agarose is added on top of the cells without using a gene frame. (iii) Samples after EPON embedding. For glass embedding, the glass coverslip is broken off the EPON disc prior to sectioning. **b** Overview pictures of *B. subtilis* 168 cells embedded on a single agarose layer (top) and as pellet (bottom) at 5000x magnification.

Light microscopy studies of bacteria commonly use thin agarose layers to immobilize cells^11,12^. If done correctly, these cells are well-aligned in a single plane, allowing large-scale quantification of phenotypic changes. We wondered whether this immobilization technique could be adapted for TEM embedding, which would solve the issue of randomly sectioned bacteria and at the same time drastically reduce the required sample volume. Using rod-shaped *B. subtilis* cells as model sample, we tested different conditions, eventually resulting in the following flat embedding procedure. As little as 50–150 µl of logarithmically growing (OD_600_ = 0.4) *B. subtilis* culture was pelleted, resuspended in 5–15 µl medium and spotted on a thin, flat layer of 1.5% agarose (Fig. 1a, Supplementary Movies 1 and 2). After evaporation of excess liquid, the immobilized cells were subjected to a standard sequence of fixation, staining, dehydration, and finally resin embedding, resulting in an EPON disc carrying the flat embedded bacteria (Supplementary Fig. 1). Some cells were washed off during the procedure, but the majority remained attached to the agarose and was successfully embedded. As shown in Fig. 1b, cells were generally well-aligned in the resulting ultrathin sections. Only five images of a single ultrathin section were sufficient to examine more than 900 individual fully longitudinally sectioned cells (5000x magnification). When we examined TEM pictures of bacteria prepared with the classical pellet embedding method, we found on average only six fully longitudinally sectioned bacteria per image (Fig. 1b). Even filamentous cell division mutants, which normally pose a particular challenge for TEM, could be efficiently sectioned longitudinally using the flat embedding protocol (Supplementary Fig. 2).

### Flat embedding applied to different bacteria

**Fig. 2:**
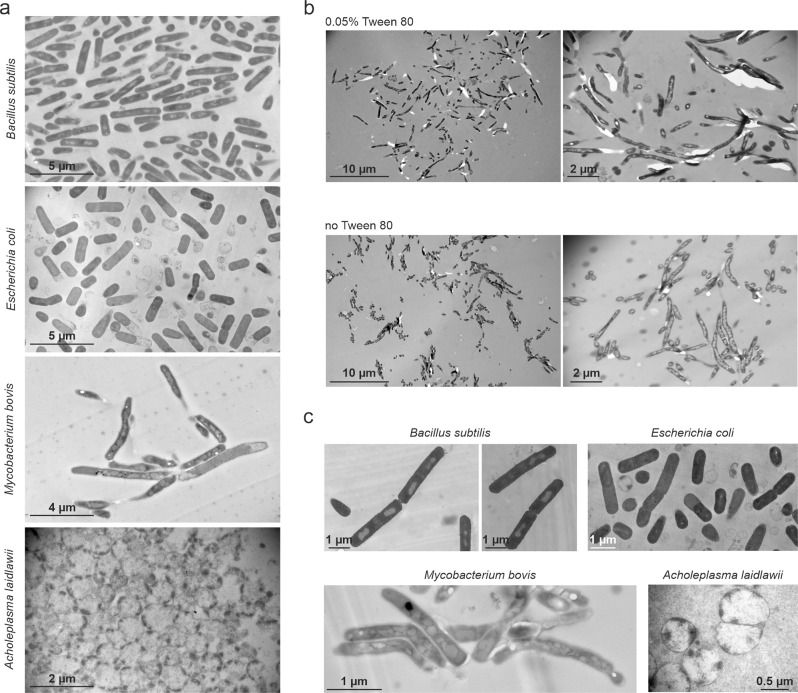
Transmission electron micrographs of flat-embedded *Bacillus subtilis, Escherichia coli, Mycobacterium bovis* BCG, and *Acholeplasma laidlawii*. **a** Bacteria embedded on a single layer of agarose. **b** Agarose sandwich approach to flat embedding of mycobacteria. *M. bovis* BCG was grown in the presence or absence of 0.05% Tween 80. **c** Bacteria embedded on carbon-coated coverslips. See also Supplementary Fig. 3.

To examine whether flat embedding is applicable to a wider range of microorganisms, we tested bacterial species with different cell surface properties. *E. coli* was chosen as representative of Gram-negative bacteria, *M. bovis* BCG as representative of bacteria with a mycolic acid-containing outer membrane, and *A. laidlawii* as a cell wall-less mycoplasma species. Both *E. coli* and *A. laidlawii* were easy to embed on agarose (Fig. 2a and Supplementary Fig. 3). However, *M. bovis* BCG was easily washed off the agarose surface during subsequent washing and fixation steps, resulting in only very few cells being left on the final sections. Typically, *M. bovis* BCG is grown in the presence of detergent (Tween 80) to reduce clumping and to facilitate microscopic observation of single cells^13,14^. However, the presence of detergent might reduce the mycobacterial capsule and affect cell morphology^13,15–20^, and we hypothesized that it might also affect the attachment of the cells to the agarose patch. However, growing *M. bovis* BCG without detergent did not improve attachment to the agarose surface. On the contrary, clumping cells detached even more readily and could not be embedded with this method. To overcome this problem, we developed an agarose sandwich approach. To this end, cells were covered with a second thin layer of agarose after spotting on the first flat agarose layer (Fig. 1a and Supplementary Movie 2). Using this approach, we were able to easily embed both detergent-treated and detergent-free cultures of *M. bovis* (Fig. 2b and Supplementary Fig. 3). White patches in *M. bovis* samples are areas where a piece of resin was removed during ultrathin sectioning. This is a typical artifact for mycobacterial TEM samples and is possibly due to their thick capsule and extracellular matrix^21–24^.

In conclusion, flat agarose patches can be used to immobilize a wide variety of bacterial cells in a single plane for longitudinal TEM sectioning.

### Flat embedding on carbon-coated glass surfaces

While flat embedding on agarose was easy and straight forward, it can be time-consuming to find the perfect plane during ultrathin sectioning. To facilitate this step, we developed an alternative embedding method by spotting cells onto a carbon-coated glass coverslip (Fig. 1a). The carbon film was applied to better visualize the bacteria during sectioning. After embedding, the glass was removed from the polymerized resin, leaving the cells very close to the surface of the EPON disc. This, and the easy localization of the cells due to the contrast of the dark carbon film greatly facilitated finding the right section plane. Since only cells and no agarose patches have to be dehydrated in this protocol, it is significantly faster at the embedding stage as well. It also eliminates the risk of artifacts caused by insufficient dehydration of the agarose film, which can complicate sectioning and produces “waves” in the sections. As shown in Fig. 2c, embedding on glass worked for all tested species and resulted in flat, clean, and nicely sectioned samples (see also Supplementary Fig. 3). However, cells detached easier from glass than from the agarose surface, resulting in considerably less cells in the final sections.

### Antibiotic mode of action studies

**Fig. 3:**
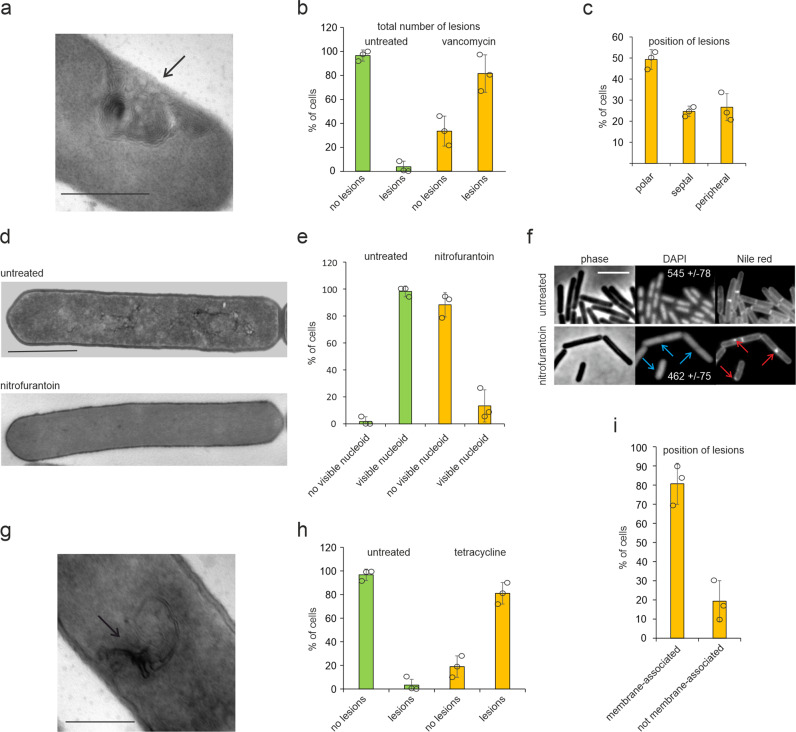
Quantification of antibiotic-induced lesions based on electron micrographs. **a** TEM image showing cell wall damage (arrow) caused by vancomycin. Scale bar 500 nm. **b** Quantification of the total number of lesions caused by vancomycin. **c** Position of cell wall lesions in vancomycin-treated cells. **d** TEM images showing deterioration of nucleoids caused by nitrofurantoin. Scale bar 1 µm. **e** Quantification of cells devoid of visible nucleoids in electron micrographs. Note that all nitrofurantoin-treated cells with visible DNA structures displayed a deteriorated nucleoid as shown in Supplementary Fig. 6. **f** Fluorescence light microscopy images of *B. subtilis* stained with the DNA dye DAPI and the membrane dye Nile red. Blue arrows indicate diffuse DNA stain. Numbers in the DAPI panels show average cell fluorescence quantified from three different data sets using the ImageJ analyze particles function. Red arrows indicate membrane patches. Cells were treated with 4x MIC of nitrofurantoin for 30 min. Scale bar 3 µm. **g** TEM image showing a lesion (arrow) caused by tetracycline. Scale bar 500 nm. **h** Quantification of total lesions in cells treated with tetracycline. **i** Quantification of different types of membrane lesions caused by tetracycline. (**b**, **c**, **e**, **h**, **i**) Cells were quantified manually from electron micrographs at 8000 to 15,000x magnification according to their phenotype (*n* ≥ 100 per condition per replicate). Error bars represent the standard deviation of three biological replicates. Circles indicate individual datapoints.

Our flat embedding method enables a quantitative approach to monitor antibiotic-induced cell damage using TEM. To demonstrate this, we counted antibiotic-induced phenotypic changes in at least 100 *B. subtilis* cells caused by a panel of well-characterized antimicrobial compounds, including vancomycin, ampicillin, daptomycin, MP196, nitrofurantoin, and tetracycline. Concentrations were used that clearly reduced the growth rate without causing extensive cell lysis (Supplementary Table 1 and Supplementary Fig. 4). After incubation of cultures with the selected antibiotic concentrations for 30 min, samples were embedded using the single layer agarose approach. Typical examples of cells exhibiting cellular aberrations that are characteristic for the individual antibiotics are shown in Supplementary Fig. 5. While ampicillin, daptomycin, and MP196 caused the expected phenotype (see legend of Supplementary Fig. 4 for details), vancomycin, nitrofurantoin, and tetracycline displayed unexpected phenotypes and were chosen for further analysis. Vancomycin, a last line of defense antibiotic for systemic Gram-positive infections, binds to the peptidoglycan precursor molecule lipid II^25^. Cells treated with this antibiotic showed characteristic cell envelope lesions that are indicative of aberrant cell wall synthesis. However, only in 32% of cells vancomycin-induced lesions occurred at the cell periphery, while in 44% of the cells, they were located at cell poles and 22% at cell division septa (Fig. 3c and Supplementary Fig. 6a). The locally increased concentration of lipid II at developing septa^26^ might explain the higher proportion of lesions occurring at new and old cell division sites, i.e., cell poles.

Nitrofurantoin is an antibiotic that is widely used against urinary tract infections since 1953, but its mechanism is still not fully understood. It is thought to damage DNA, RNA, proteins, and other macromolecules by a mechanism involving oxidative damage^27^. Interestingly, in our TEM images 74% of cells treated with nitrofurantoin seemed to entirely lack a visible nucleoid, whereas the other 26% showed condensed remnants of chromosomes (Fig. 3c, e and Supplementary Fig. 6b). To confirm this finding, live cells were stained with the fluorescent DNA dye DAPI and examined by fluorescence light microscopy. Already after 5 min of treatment cells started to show condensed nucleoids (Supplementary Fig. 7) and after 30 min the DAPI signal became completely diffuse (Fig. 3f). The overall DAPI signal inside these cells was decreased by 26% (*p* < 0.001) compared to the untreated control (Fig. 3f). The TEM images also showed accumulation of small membrane vesicles (Supplementary Fig. 5). In line, 50% of cells also showed fluorescent membrane patches when stained with the membrane dye Nile red (Fig. 3f). Both DNA and lipids are sensitive to oxidative damage^28–30^ and our results corroborate the current model of nitrofurantoin action.

The commonly used antibiotic tetracycline is known to inhibit protein biosynthesis by blocking the binding of aminoacyl-tRNA to the ribosome^10,31^. Interestingly, 90% of tetracycline-treated cells exhibited cellular lesions in the TEM images reminiscent of membrane invaginations (Fig. 3g, h). The majority of these (69%) were visibly membrane-associated (Fig. 3I and Supplementary Fig. 6c). These results may suggest that tetracycline does not only target the ribosome but also affects the bacterial cell membrane.

### Tetracycline is a membrane-active compound

**Fig. 4:**
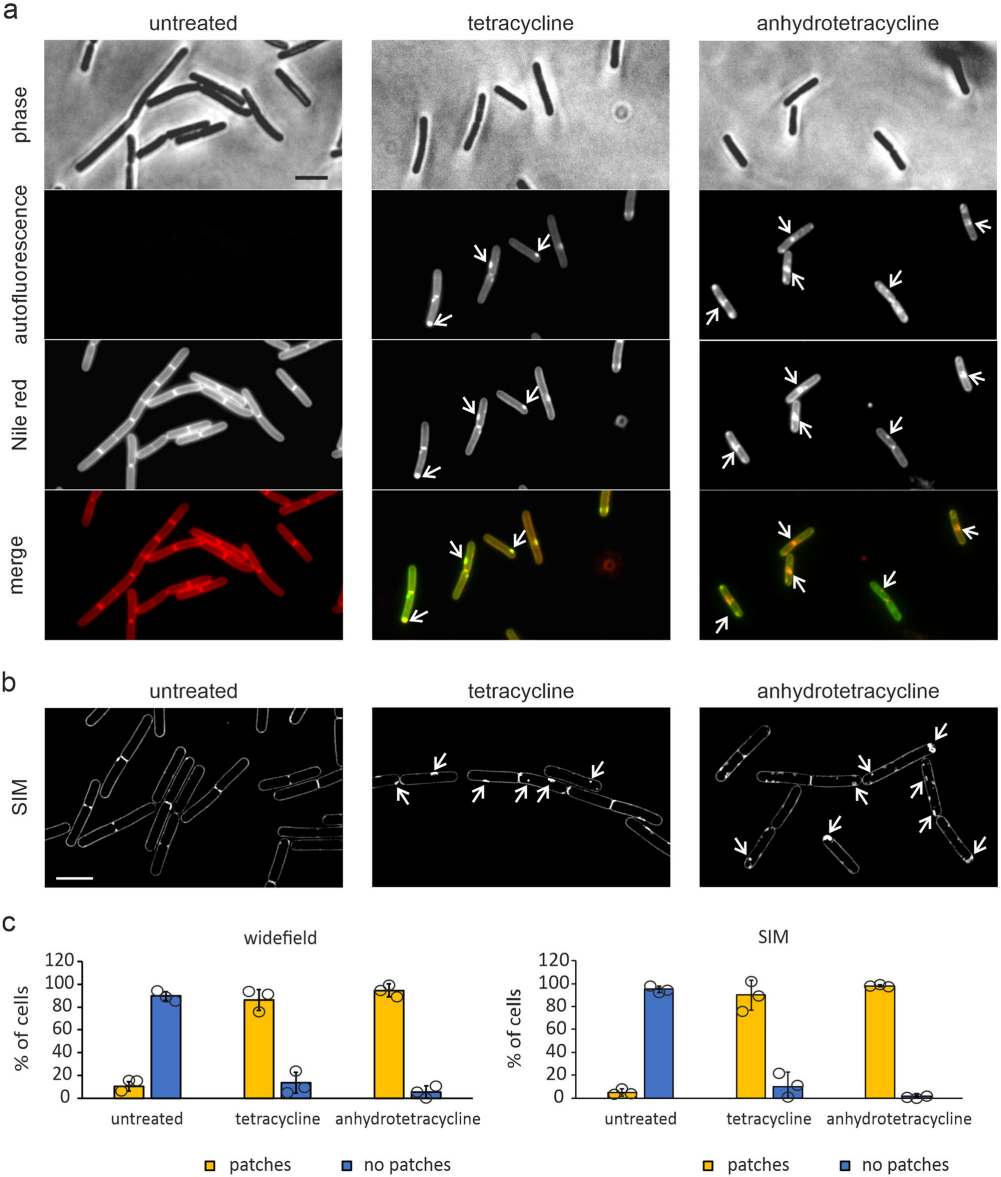
Tetracycline targets the cytoplasmic membrane. **a** Fluorescence microscopy images of cells treated with either tetracycline or anhydrotetracycline for 30 min. Both antibiotics display green autofluorescence allowing direct localization of the compound. Cell membranes were stained with Nile red. Arrows indicate fluorescent membrane patches coinciding with accumulation of the respective antibiotic. **b** SIM microscopy images of cells treated with either tetracycline or anhydrotetracycline for 30 min. Membranes were stained with Nile red. Arrows indicate membrane staples or invaginations. Scale bars 2 µm. **c** Quantification of cells showing membrane patches from widefield (**a**) and SIM (**b**) images. Images were quantified manually. Sample size was ≥100 individual cells per condition for widefield and ≥50 individual cells per condition for SIM. Error bars show standard deviation of the mean of three replicate experiments. Circles indicate individual datapoints.

To investigate the effect of tetracycline on bacterial cell membranes in more detail, we tested whether we could observe membrane deformations with fluorescence microscopy using the membrane dye Nile red. As shown in Fig. 4a, tetracycline indeed caused aberrant, highly fluorescent membrane patches in 93% of cells (Supplementary Fig. 8). We were able to localize the antibiotic directly due to its green autofluorescence, which appeared to overlap with Nile red-stained membrane foci (Fig. 4a, c). This irregular green fluorescence membrane staining was also observed in cells that were not stained with Nile red (Supplementary Fig. 8), indicating that it is not a fluorescence bleed-through artifact from the bright membrane dye.

The TEM images suggested that the highly fluorescent Nile red patches are likely caused by the accumulation of extra membrane material due to membrane invaginations^32–34^ (Fig. 3g and Supplementary Figs. 5 and 6). To confirm this, we increased the fluorescence microscopy resolution by employing structured illumination microscopy (SIM). This revealed clear membrane invaginations after treatment with tetracycline (Fig. 4b, c). Additional evidence for invaginations was provided by accumulation of GFP-tagged AtpA in specific sites in the cell. This F1F0 ATP synthase subunit is a regularly distributed membrane protein that is insensitive to disturbance of most membrane parameters but does show an increased fluorescence signal when a double membrane is present^32,35^. Exactly this phenotype was observed with tetracycline (Supplementary Fig. 9).

The tetracycline analog anhydrotetracycline is broadly applied in molecular genetics as inducer of Tet repressor-based gene expression systems^36,37^, since it is widely believed not to inhibit translation or bacterial growth^38^. Interestingly, incubation of *B. subtilis* cells with anhydrotetracycline also caused fluorescent Nile red foci that appear to be caused by membrane invaginations (Fig. 4a–c). These results confirmed our observations by TEM (Supplementary Figs. 5 and 10), suggesting that tetracycline affects the bacterial cell membrane independently from the inhibition of protein translation.

### Tetracycline affects membrane protein localization

**Fig. 5:**
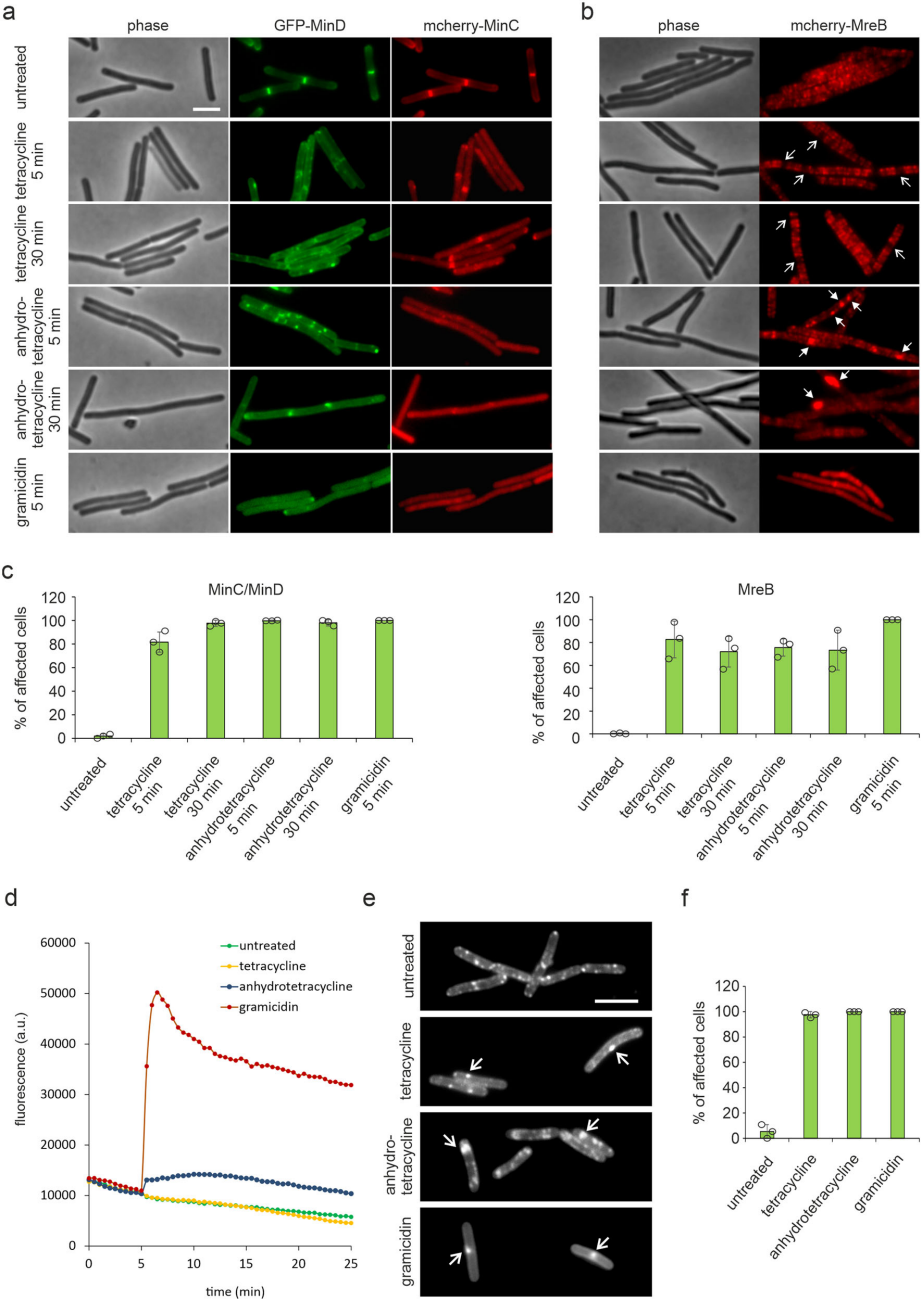
Tetracyclines delocalize membrane proteins. Effects of tetracycline, anhydrotetracycline, and gramicidin (positive control) on protein localization. *B. subtilis* LB318 (168 *amyE::spc mgfp-minD aprE::cat mcherry-minC*) **a** and TNVS205 (168 *aprE::cat mcherry-mreB*) **b** were treated with 2 µg/ml tetracycline, 2 µg/ml anhydrotetracycline, or 1 µg/ml gramicidin, respectively. Arrows indicate abnormalities in protein localization patterns. Scale bars 2 µm. **c** Quantification of microscopy images pertaining to (**a**) and (**b**). MinC localization depends on MinD resulting in MinC always being affected when MinD is delocalized. Hence, the numbers of affected cells are the same for both proteins and only one graph is shown. A MinCD-expressing cell was counted as affected, when it lost its typical septal/polar localization pattern by displacement of the fluorescence signal into the cytosol and/or membrane patches. MreB-expressing cells were counted as affected, when the regular MreB localization was disturbed by gaps (typical for tetracycline), patches (typical for anhydrotetracycline), or displacement of the fluorescence signal into the cytosol (typical for gramicidin and partially anhydrotetracycline). Images were quantified manually. Sample size was ≥100 individual cells per condition. Error bars show standard deviation of the mean of three replicate experiments. Circles indicate individual datapoints. **d** Effects of tetracycline, anhydrotetracycline, and gramicidin on the membrane potential of *B. subtilis* 168 measured with DiSC(3)5. An exemplary graph out of three biological replicates is shown. **e** Effects of tetracycline, anhydrotetracycline, and gramicidin on fluid membrane microdomains of *B. subtilis 168* cells stained with the fluid lipid domain dye DiIC12. Arrows indicate abnormal membrane domain stains. Scale bar 2 µm (**f**) Quantification of microscopy images pertaining to (**e**). Images were quantified manually. Cells were counted as affected, when the regular DiIC12 staining pattern deviated from the control (regular distribution, regular size, typically 6–15 spots per cell) by accumulation of the dye in irregular, large, and/or less than 6 foci). Sample size was ≥100 individual cells per condition. Error bars show standard deviation of the mean from three replicate experiments. Circles indicate individual datapoints.

To test whether tetracycline functionally disturbs the cell membrane, we examined the localization of three peripheral membrane proteins that are known to be affected by membrane depolarization, MinC, MinD, and MreB^39,40^. MinD interacts with MinC to form a complex that inhibits initiation of cell division at the cell poles^41^. Using a strain that expresses a GFP fusion to MinD and an mcherry fusion to MinC^42^, we observed that the localization of both proteins was severely disturbed by both tetracycline and anhydrotetracycline after only 5 min of treatment (Fig. 5a, c). MreB is an actin homolog that forms dynamic polymers along the lateral membrane and coordinates lateral cell wall synthesis^43^. Tetracycline slightly affected localization of MreB and caused gaps in the normally regular localization pattern of this protein (Fig. 5b, c). Anhydrotetracycline caused a much more dramatic effect and completely delocalized MreB resulting in diffuse fluorescence signal and large local clusters (Fig. 5b).

### Tetracycline does not dissipate the membrane potential

Since the localization of MinC, MinD, and MreB depends on the membrane potential, we wondered whether tetracycline depolarizes the cell membrane. This was tested using the voltage-sensitive probe DiSC(3)5, which accumulates in bacterial cells in a membrane potential-dependent manner^11^. As shown in Fig. 5d, no depolarization of the cell membrane was observed, even after 30 min of incubation. Anhydrotetracycline caused partial membrane depolarization. The latter effect was not due to the presence of a subset of cells that had lost their membrane potential (Supplementary Figs. 11–13). These results show that tetracycline disturbs the bacterial cell membrane by a mechanism that is unrelated to membrane permeabilization.

### Tetracycline disturbs membrane organization

Bacteria can contain specific membrane regions of increased fluidity called RIFs^43,44^. RIFs contain fluidizing lipid species, e.g., with short, branched, or unsaturated fatty acid chains. Since insertion of a membrane anchor into a lipid bilayer is facilitated in a more fluid environment, RIFs are enriched in certain peripheral membrane proteins^43,45,46^. MreB is associated with RIFs^43^ and its observed delocalization could be an indication that tetracycline affects these lipid domains. RIFs can be visualized with the fluidity-sensitive dye DiIC12^12,43^. As shown in Fig. 5e, f, tetracycline, and especially anhydrotetracycline, affected the distribution of RIFS in a different manner than the membrane-depolarizing peptide gramicidin. Thus, the delocalization of MinCD and MreB is likely a consequence of the distortion of lipid organization by the tetracyclines, and not due to membrane depolarization.

### Membrane activity is independent of ribosome inhibition

Several observations suggested that tetracycline directly targets the bacterial cell membrane independently from inhibition of ribosomes. Firstly, the antibiotic visibly accumulated in the membrane lesions observed by fluorescence microscopy (Fig. 4a). Secondly, the localization of membrane proteins was affected after a short treatment time (Fig. 5). And thirdly, membrane deformations caused by tetracycline are largely similar to those of anhydrotetracycline (Figs. 4 and 5, Supplementary Figs. 5, 8, 10). As an additional control, we tested the effects of the translation inhibitors chloramphenicol and kanamycin on membrane organization. Neither chloramphenicol nor kanamycin caused membrane invaginations, affected the localization of MinCD and MreB, or affected RIFs (Supplementary Figs. 14–17).

**Fig. 6:**
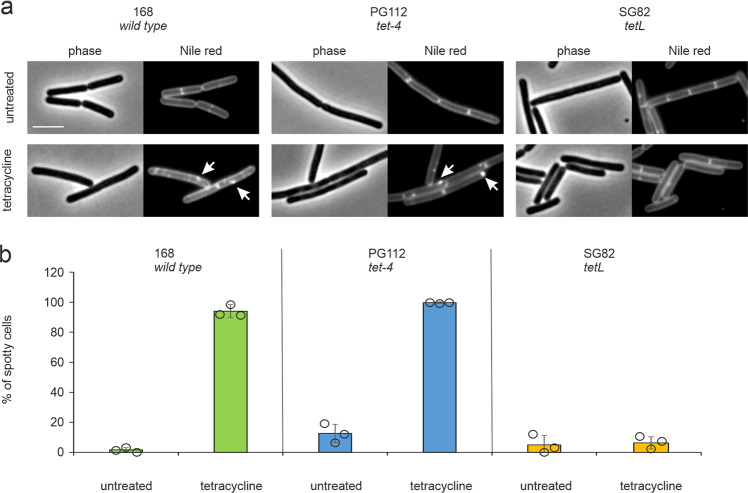
Tetracycline affects the membrane independently of ribosome inhibition. Strain PG112 carries the *tet-4* mutation, a point mutation in the ribosomal protein S10 that renders ribosomes insensitive to tetracycline. Strain SG82 carries the *tetL* tetracycline efflux pump. Cells were treated with 1x MIC of tetracycline (16 µg/ml for PG112, 100 µg/ml for SG82) for 30 min prior to membrane staining with Nile red and microscopy. See Supplementary Fig. 18 for titration of tetracycline concentrations. **a** Microscopy images of Nile red-stained cells. Scale bar 2 µm. **b** Quantification of microscopy images showing the percentage of spotty (= membrane-stressed) cells. Quantification was done manually. Sample size was ≥100 individual cells per condition. Error bars show standard deviation of the mean from three replicate experiments. Circles indicate individual datapoints.

Finally, we analyzed two different tetracycline-resistant strains, the *tet-4* point mutation in the ribosomal protein S10, which reduces the tetracycline sensitivity of the ribosome^47,48^, and a strain containing the *tetL* resistance cassette, which encodes the TetA tetracycline transporter and confers high-level tetracycline resistance^49^. If the effects of tetracycline on the membrane are a consequence of ribosome inhibition, they should be absent in both the *tet-4* and *tetL* mutant. As shown in Fig. 6 and Supplementary Fig. 18, membrane distortions were still clearly visible in the *tet-4* mutant, indicating that the interaction of tetracycline with ribosomes is not required for its membrane activity. In contrast, the *tetL* mutant showed no membrane lesions, which makes sense since TetA is an efflux pump that removes tetracycline from the membrane^49^. As a control we used anhydrotetracycline, which is not affected by either resistance mechanism. As expected, anhydrotetracycline induced membrane lesions in all strains (Supplementary Fig. 19).

## Discussion

Here we described a new method for embedding bacterial cells in a single layer to facilitate observation by TEM. This technique enabled us to observe high numbers of longitudinally cut bacterial cells and easily quantify antibiotic-induced phenotypes. Flat embedding is straight forward, does not require any further technology or resources, and can be adapted in any laboratory equipped for TEM. However, two points have to be taken into consideration when applying this technique. Firstly, embedding on agarose requires careful dehydration, since residual water will lead to infiltration artifacts that either jeopardize ultrathin sectioning or, if sectioning is still possible, appear as strong ‘wavy’ background in the final sections. Insufficient dehydration might also lead to crooking of the agarose patch after overnight incubation with the EPON resin, defying the purpose of flat embedding. Therefore, dehydration steps should not be shortened and the agarose layer must be thin, especially for the sandwich approach. In this respect, flat embedding on carbon-coated glass surfaces is clearly an advantage, since in this case only the cells need to be dehydrated. Secondly, since all cells are aligned in one plane in both agarose and glass methods, ultrathin sectioning requires an experienced person in order to hit the resin block at a perfectly perpendicular angle to the cells. While finding the right plane is easier in glass-embedded samples due to the carbon film, it also requires higher precision and care, since the almost perfect alignment of cells limits the tolerance for failed sectioning attempts. Ultimately, we found the sandwich approach to be the easiest and most versatile protocol. It prevents the detachment of bacteria during washing, which greatly facilitates sample handling. Sandwich embedding worked well for all species, even *M. bovis*, which easily detached from surfaces during handling, and it facilitates working with pathogenic strains, since they are safely enclosed between the agarose layers.

Our flat embedding method revealed a previously unknown antibacterial mechanism of tetracycline, which is independent from its ability to inhibit the ribosome. The membrane-distorting effect of tetracycline resulted in the complete delocalization of the cell division-regulatory protein couple MinCD, which could explain an earlier observation that certain *B. subtilis* cell division mutants are hypersensitive to tetracycline, a phenomenon that was also independent from the ribosome-inhibiting activity of tetracycline^50^. Tetracycline also disturbed the localization of the cytoskeletal protein MreB and it is reasonable to assume that more membrane proteins will be affected, which would substantially impact the viability of cells. This additional activity of tetracycline also explains why the ribosomal *tet-4* mutation confers much lower levels of tetracycline resistance (MIC = 16 µg/ml) than the *tetL* resistance cassette that encodes for an efflux pump (MIC = 100 µg/ml). There is still a strong bias against membrane-targeting antibiotics, since they have a reputation to be unspecific and generally toxic. The fact that such an established antibiotic as tetracycline has been “secretly” targeting the bacterial cell membrane for such a long time, underlines that the bacterial cell membrane can be successfully targeted without major side effects on human cells.

Membrane activity has not been clearly shown for tetracycline before. An early study showed that tetracycline caused leakage of small molecules from *E. coli* cells, but only at 100 to 400-times the concentrations necessary to completely inhibit translation, leaving the relevance of these findings unclear^51^. The tetracycline analog anhydrotetracycline has previously been suspected to target the cell membrane and to cause depolarization. This was based on the fact that anhydrotetracycline causes cell lysis in *E. coli*^52^. Our data now show that anhydrotetracycline does indeed directly affect the cell membrane, however, it does not kill by membrane depolarization. Our findings have significant implications for the use of anhydrotetracycline as inducer of gene expression, which is widely advertised to not have antibacterial activity^38^. In fact, we have shown that anhydrotetracycline has an even higher antibiotic activity than tetracycline (Supplementary Table 1).

It has been shown that chelocardin, another member of the tetracycline group of antibiotics, inhibits translation but at higher concentrations also causes membrane stress^53^. Very recently, a similar observation was made for anhydrotetracycline^54^. Both compounds are often referred to as “atypical tetracyclines”, which are characterized by being bactericidal. The typical tetracyclines, such as oxytetracycline and tetracycline itself, are bacteriostatic and assumed to only target the ribosome^10^. Our study now shows that both groups share membrane distortion as an overarching feature of their antibacterial activity.

How exactly tetracycline affects the cell membrane remains to be investigated. It has been proposed that due to their rather hydrophobic core structure, tetracyclines remain in the cytoplasmic membrane for a relatively long time before they translocate into the cytosol^10,55^. In fact, the clear membrane fluorescence signal observed with both tetracycline and anhydrotetracycline supports this hypothesis. It is reasonable to assume that the same chemical properties that retain these molecules in the membrane also promote bilayer distortion. Tetracycline is a large molecule with a bulky structure, which is likely to disturb the organization of the lipid bilayer. Anhydrotetracycline possesses a methyl group instead of the hydroxyl group, which stimulates interaction with the hydrocarbon core of the lipid bilayer^56^. This may explain why anhydrotetracycline has a more severe effect on membrane organization.

From oxytetracycline, which was the first tetracycline to become commercially available in 1950, to doxycycline, which is one of the most commonly prescribed antibiotic drugs today, tetracyclines are widely used in human and veterinary medicine. Despite this heavy use, target-based resistance mutations against tetracycline occur slowly, which has been attributed to the fact that ribosomes are encoded by multiple genes^57^. However, resistance against other ribosome inhibitors like streptomycin is frequently observed^58^. Therefore, an alternative explanation for the low resistance development against tetracyclines could be that they have a second target, the cell membrane, for which it is generally difficult to obtain suppressor mutations^59^. Developing tetracyclines with enhanced membrane effects could be a desirable strategy to combat bacterial infections, since membrane-active bactericidal compounds are often also effective against persister cells, which are an increasing problem in the clinic^60–62^. Finally, our results underscore the emerging realization that multi-target antibiotics are most successful in clinical use^57^.

## Methods

### Antibiotics

Gramicidin, vancomycin, ampicillin, nitrofurantoin, tetracycline, anhydrotetracycline, kanamycin, and chloramphenicol were purchased from Sigma-Aldrich in the highest available purity. Daptomycin was purchased from Abcam. MP196 was synthesized by solid-phase synthesis as described previously^63^. Gramicidin, nitrofurantoin, anhydrotetracycline, and MP196 were dissolved in sterile DMSO. Vancomycin, ampicillin, kanamycin, and daptomycin were dissolved in sterile water. Tetracycline and chloramphenicol were dissolved in ethanol.

### Strain construction

A complete strain list can be found in Supplementary Table 2. For construction of *B. subtilis* TNVS205 (*aprE::cat-Pspac-mcherry-mreB*) the *mreB* gene was amplified using the primer pair TerS397/TerS400 and the plasmid pAPNC213-cat^64^ was amplified with the primer pairs TerS398/TerS337 and TerS338/135. The resulting PCR products were subjected to a three-fragment Gibson assembly reaction^65^ resulting in plasmid pTNV86, which was transformed into *B. subtilis* 168 using a standard starvation protocol^66^, resulting in TNVS205. See Supplementary Table 3 for primer sequences.

### Minimal inhibitory concentration (MIC)

Minimal inhibitory concentrations were determined in a serial dilution assay as described in^67^. Briefly, lysogeny broth (LB) was supplemented with different antibiotic concentrations and inoculated with 5 × 10^5^ CFU/ml of *B. subtilis* 168^68^. Cells were grown at 37 °C under steady agitation for 16 h. The lowest antibiotic concentration inhibiting visible bacterial growth was defined as MIC. The MIC of daptomycin was tested in presence of 1.25 mM CaCl_2_. MICs were performed in duplicates yielding identical results.

### Growth experiments

*B. subtilis* 168 was aerobically grown in LB. Overnight cultures were diluted to an OD_600_ of 0.05 and allowed to grow until an OD_600_ of 0.4 prior to addition of antibiotics at different MIC multiples. Growth was monitored for 8 h using a Biotek Synergy MX plate reader equipped with Gen5 software. Concentrations leading to a reduced growth rate without causing massive cell lysis within the first 30 min of antibiotic exposure were chosen for electron microscopy. Daptomycin requires the presence of 1.25 mM CaCl_2_. Addition of CaCl_2_ did not affect growth of *B. subtilis*. Experiments were performed in duplicates.

### Growth conditions for TEM experiments

TEM experiments were performed in triplicates. *B. subtilis* 168 and *E. coli* MG1655 were grown in LB. *B. subtilis* MW18 was grown in the presence of 50 µg/ml spectinomycin and 0.5 mM isopropyl β-D-1-thiogalactopyranoside (IPTG) overnight and diluted 1:100 into antibiotic-free medium containing 0.5 mM IPTG for the embedding experiment. *M. bovis* BCG Tice^69^ was grown in 7H9 medium (Difco) supplemented with Middlebrook albumin/dextrose/catalase supplement (BD Biosciences), and 0.05% Tween 80. When *M. bovis* BCG was to be observed without detergent treatment, cultures were washed and resuspended in fresh medium without Tween two days prior to the embedding procedure. *A. laidlawii* PG-8A was grown in modified PPLO medium (1.41% PPLO broth (BD Biosciences), 0.15% TC Yeastolate (Difco), 1.4% glucose, 20% horse serum, 1000  U/ml penicillin G (Sigma-Aldrich)). All cultures were maintained at 37° C under continuous shaking. After reaching mid-logarithmic growth phase, 50 µl of cells were withdrawn, pelleted by centrifugation (16,000x*g*, 2 min), and resuspended in 5 µl medium. For antibiotic treatment, *B. subtilis* 168 was aerobically grown in LB to an OD_600_ of 0.3 and subsequently treated with either 32 µg/ml valinomycin, 1 µg/ml vancomycin, 1 µg/ml ampicillin, 16 µg/ml MP196, 0.5 µg/ml daptomycin, 32 µg/ml nitrofurantoin, 2 µg/ml tetracycline, 2 µg/ml anhydrotetracycline, or left untreated as control. After 30 min of antibiotic treatment, 50–150 µl of sample were pelleted by centrifugation (16,000x*g*, 2 min), and resuspended in 5–15 µl fresh LB. Higher cell densities resulted in less effective alignment of cells in the final sections.

### Flat embedding of bacteria on agarose

Five to fifteen microliter of the cell suspensions were spotted on 0.25 mm thick 1.5% agarose films (thickness was controlled using Gene Frame AB0576, ThermoFischer Scientific, see Supplementary Movie 1 for preparation). Excess liquid medium was allowed to evaporate under a slight air flow in a clean air bench. This process is derived from a standard immobilization technique for live cell fluorescence microscopy of bacteria. If a small amount of cell material is spotted on the surface, cells will align on the agarose matrix as the excess medium evaporates. This can easily be monitored by eye as the dome of the liquid droplet disappears, which normally takes 30–60 sec and is enough to stop the bacteria from excessive swimming allowing the alignment necessary for flat embedding. Since the agarose patch itself is well-hydrated at this point, the cells themselves do in fact not dry out during this process and, if appropriately covered to prevent further dehydration of the agarose patch, grow and divide normally for many hours. A 10 µl drop of sample created an area large enough to produce at least five individual blocks for sectioning. Volumes less than 5 µl still resulted in one or two blocks, making it well possible to work with initial culture volumes of less than 50 µl. For our antibiotic study we chose to spot 10 µl to have more material in case the antibiotic-treated cells would attach less efficiently to the agarose layer. For *M. tuberculosis*, which in general did not attach very well to agarose, we used 15 µl while for the other non-antibiotic treated bacterial samples 5 µl were sufficient. Spotting volumes higher than 15 µl or using higher concentrated samples did not further increase the number of longitudinally cut cells and in fact compromised their alignment. Agarose patches were transferred to aluminum dishes and kept free-floating (not sticking to the bottom of the dish to allow optimal diffusion of solutions into the agarose film) in the respective solutions with the cell samples facing upwards during all fixation, washing, and dehydration steps. Mounted cells were fixed in 5% glutaraldehyde (Merck) in 0.1 M cacodylate (Sigma-Aldrich) buffer (pH 7.4) for 20 min. Samples were subsequently washed three times with 0.1 M cacodylate, pH 7.4 for 5 min each, followed by incubation in 1% OsO_4_ (EMS)/1% K_4_Ru(II)(CN)_6_ (Sigma-Aldrich) for 30 min. Samples were then washed three times with ultrapure water for 5 min each. Dehydration was performed in an incubation series with rising ethanol (Merck) concentrations as follows: 5 min 30% ethanol, 5 min 50% ethanol, 2 × 15 min 70% ethanol, 1 h 80% ethanol, 15 min 90% ethanol, 15 min 96% ethanol, 15 min 100% ethanol, 30 min 100% ethanol, water-free. Water-free ethanol was prepared by adding 2 ml acidulated 2,2-dimethoxypropan (two drops of 37% HCl ad 100 ml 2,2-dimethoxypropan (Sigma-Aldrich)) to 100 ml ethanol absolute (Merck). Cells were then incubated for 30 min in a 1:1 mixture of EPON and propylene oxide (EMS), followed by 30 min incubation in 2:1 EPON / propylene oxide. All fixation, washing, and dehydration solutions were gently and slowly added starting from the side of the agarose patch in order not to wash off the spotted cells. Agarose patches were transferred to fresh aluminum dishes and covered with fresh EPON. Samples were left at room temperature overnight and then incubated at 65 °C for at least 36 h. EPON was prepared by mixing 48 g glycid ether (Serva) with 32 g dodecenylsuccinic anhydride (Serva) and 20 g methyl nadic anhydride (Serva). Components were mixed for 10 min prior to addition of four times 650 µl benzyldimethylamine (Serva) and mixed for an additional 15 min. EPON aliquots were kept at −20 °C until use. EPON solutions should not be kept for longer than 1 week prior to embedding to avoid infiltration artifacts. For flat embedding, it turned out that EPON prepared with glycid ether is superior to EPON prepared with EMbed (EMS), since the latter results in less flexible EPON disks, which are more difficult to cut when selecting areas of interest for mounting.

### Classical pellet embedding

For pellet embedding, a 50 ml culture was harvested and the resulting pellet was fixed and dehydrated as above, with the only difference that cell pellets were incubated with the different fixation, washing, and dehydration solutions in glass vials under slow agitation. Fixed and dehydrated cell pellets were embedded in EPON using standard conical tip capsules.

### Flat embedding of bacteria on glass slides

Bacteria were grown and concentrated as described above. Concentrated cell suspensions (10 µl) were spotted on glass coverslips that were coated with a thin carbon film as contrast agent to facilitate correct positioning of the EPON block during sectioning. Carbon-coated glass coverslips are compatible with light microscopy and have been used in correlative light electron microscopy studies previously^70,71^. Cells were mounted as described above and subsequently fixed in 5% glutaraldehyde/0.1 M cacodylate (pH 7.4) for 20 min. Samples were washed three times with 0.1 M cacodylate (pH 7.4) for 5 min each, followed by incubation in 1% OsO_4_/1% K_4_Ru(II)(CN)_6_ for 30 min. Since in this preparation procedure only the cells themselves and no agarose films need to be dehydrated, shorter dehydration times are possible. After washing the samples three times with ultrapure water (5 min each), dehydration was performed as follows: 5 min 30% ethanol, 5 min 50% ethanol, 5 min 70% ethanol, 30 min 80% ethanol, 15 min 90% ethanol, 15 min 96% ethanol, 5 min 100% ethanol, 15 min 100% ethanol, water-free, 30 min 50% EPON/50% water-free ethanol. Slides were then transferred to fresh aluminum dishes and further prepared as described above.

### Sandwich embedding of *M. bovis* BCG

While *M. bovis* BCG aligned well on agarose, it was prone to subsequently being washed off the surface, which was effectively prevented by enclosing it in an agarose sandwich. To this end, 10 µl of cells were spotted on an agarose patch as described above. Of note, sandwich embedding allows using volumes as small as 1 µl, since no cells are lost during preparation. However, spotting less than 10 µl in our hands resulted in less clear osmium/ruthenium stains making it difficult to section these samples. After drying, the sample was covered with a second thin layer of 1.5% agarose. Low melting agarose did not give a stable and flat layer, making standard agarose superior for this method. In order not to induce a heat shock, the agarose solution was allowed to cool down to ~50 °C prior to applying it to the sample. The fresh agarose spot was immediately covered by a glass coverslip to produce a thin and flat surface. Immediately placing a small weight (e.g., a half-full 50 ml falcon tube face down) on top of the glass coverslip resulted in a thinner agarose layer, which greatly facilitates dehydration of the samples. After ~1 min the weight was removed and the coverslip was gently slid off the agarose, resulting in a flat and stable agarose sandwich (see Supplementary Movie 2 for preparation). The sandwich samples were further processed like normal agarose-embedded samples as described above.

### Electron microscopy

Regions of interest were selected by observing the EPON-embedded bacterial layer under a light microscope prior to mounting on EPON blocks for thin sectioning. Ultrathin sections (∼80 nm) were cut parallel to the bacterial layer, collected on single-slot, Formvar-coated copper grids, and subsequently counterstained with uranyl acetate (Ultrostain I, Laurylab) and lead citrate (Reynolds) in a Leica EM AC20 ultrastainer. Bacteria were imaged using a JEOL 1010 transmission electron microscope at an electron voltage of 60 kV using a side-mounted CCD camera (Modera, EMSIS) and iTEM software.

### Fluorescence light microscopy

All fluorescence microscopy experiments were performed in biological triplicates. All strains were aerobically grown in LB until an OD_600_ of 0.4 prior to antibiotic treatment. For Nile red staining *B. subtilis* 168 was treated with 2 µg/ml tetracycline, 2 µg/ml anhydrotetracycline, 15 µg/ml chloramphenicol, or 3 µg/ml kanamycin for 30 min followed by membrane staining with 0.5 µg/ml Nile red for 1 min. For DAPI staining *B. subtilis* 168 was treated with 32 µg/ml nitrofurantoin for 5, 15, 30, or 60 min, respectively, followed by staining of the chromosome with 1 µg/ml DAPI for 1 min. *B. subtilis* LB318 (168 *amyE::spc mgfp-minD aprE::cat mcherry-minC*)^42^ was grown in the presence of 0.1% xylose to induce expression of *mgfp-minD* and 0.1 mM IPTG to induce expression of *mcherry-minC*. TNVS205 (168 *aprE::cat mcherry-mreB*) was grown in the presence of 0.3 mM IPTG to induce expression of *mcherry-mreB*. *B. subtilis* LB318 and TNVS205 were treated with 2 µg/ml tetracycline, 2 µg/ml anhydrotetracycline, or 1 µg/ml gramicidin, respectively. Note that LB318 carries both a chloramphenicol and a kanamycin resistance cassette and TNVS205 carries only chloramphenicol resistance. Concentrations of chloramphenicol and kanamycin were 15 and 3 µg/ml, respectively, for non-resistant strains, and 20 and 10 µg/ml for strains carrying the respective resistance marker(s), which corresponds to double the selection concentration. Samples were observed under the microscope after 5 and 30 min of antibiotic treatment. Staining with DiSC(3)5 was carried out as described by te Winkel et al.^11^ followed by treatment with 2 µg/ml tetracycline, 2 µg/ml anhydrotetracycline, or 1 µg/ml gramicidin, respectively. Samples were examined after 5 and 30 min of antibiotic staining. DiIC12 staining was carried out as described in Müller et al.^45^. All microscopy samples were spotted on a thin film of 1.2% agarose^11^ and examined with a Nikon Eclipse Ti equipped with a CFI Plan Apochromat DM 100x oil objective, an Intensilight HG 130 W lamp, a C11440-22CU Hamamatsu ORCA camera, and NIS elements software. Images were analyzed using ImageJ (National Institutes of Health).

### Structured Illumination Microscopy (SIM)

Samples were prepared as for fluorescence light microscopy. Coverslips were coated with poly-dopamine to reduce background fluorescence by preventing binding of the membrane dye to the glass surface^11^. Cells were imaged with a Nikon Eclipse Ti N-SIM E microscope setup equipped with a CFI SR Apochromat TIRF 100x oil objective (NA1.49), a LU-N3-SIM laser unit, an Orca-Flash 4.0 sCMOS camera (Hamamatsu Photonics K.K.), and NIS elements Ar software. Images were analyzed using ImageJ (National Institutes of Health).

### Spectroscopic membrane potential measurements

Cells were cultured as for microscopy experiments and transferred to a pre-warmed 96-well plate after reaching an OD_600_ of 0.4. DiSC(3)5 measurements were carried out as described by te Winkel et al.^11^ in triplicates. Cells were treated with 2 µg/ml tetracycline, 2 µg/ml anhydrotetracycline, and 1 µg/ml gramicidin. Measurements were taken every 30 sec over a total of 30 min in a Biotek Synergy MX plate reader equipped with Gen5 software. Kanamycin (3 µg/ml) and chloramphenicol (15 µg/ml) were also tested but had no effect on the membrane potential (Supplementary Fig. 20a). Antibiotics were tested for an effect on DiSC(3)5 fluorescence in solution to control for interference with the dye but no change in DiSC(3)5 fluorescence was observed (Supplementary Fig. 20b).

### Statistics and reproducibility

All experiments were performed in biological triplicates. Experiments involving the counting of phenotypes were performed manually according to the specific phenotypic variations defined in the respective figure legends. For quantification of phenotypes from TEM images, only fully longitudinally sectioned cells were counted. For quantification from fluorescence microscopy images, only cells in focus were included. All bar charts that show counted phenotypes depict mean number of cells with standard deviation from three replicates. A minimum of 100 cells were counted per replicate per condition with the exception of SIM, which has a much smaller field of view. Here, we counted 50 cells per condition per replicate. All quantifications and graphs were done in Excel (MS Office 365 ProPlus). Figures were arranged in Corel Draw Graphics Suite 2019.

### Reporting Summary

Further information on research design is available in the Nature Research Reporting Summary linked to this article.

## Supplementary information

Supplementary Information

Description of Supplementary Files

Supplementary Movie 1

Supplementary Movie 2

Supplementary Data 1

Reporting Summary

## Data Availability

All data necessary to interpret the results of our study are included in the article and Supplementary Information. Quantification and statistics of microscopy are included in the Supplementary data files. Further information and raw image files may be obtained on reasonable request from wenzelm@chalmers.se.
