# Supplementary material for: A flat embedding method for transmission electron microscopy reveals an unknown mechanism of tetracycline
Source: Commun Biol. 2021 Mar 8;4:306. doi: 10.1038/s42003-021-01809-8 (PMC7940657; doi:10.1038/s42003-021-01809-8)
Supplement: Supplementary file 1 — Supplementary Information [file 42003_2021_1809_MOESM1_ESM.pdf]

Supplementary Information

A flat embedding method for transmission electron microscopy reveals an unknown mechanism of tetracycline

Michaela Wenzel, Marien P. Dekker, Biwen Wang, Maroeska J. Burggraaf, Wilbert Bitter, Jan R. T. van Weering, Leendert W. Hamoen

Supplementary Table 1: Minimal inhibitory concentrations of antimicrobial compounds against *B. subtilis* 168.

Supplementary Table 2: Bacterial strains used in this study.

Supplementary Table 3: Primer sequences.

Supplementary Figure 1: Example of a polymerized EPON disc after flat embedding.

Supplementary Figure 2: Flat embedding of filamentous cells.

Supplementary Figure 3: Details of flat-embedded cells.

Supplementary Figure 4: Growth of *B. subtilis* 168 after treatment with different antibiotic concentrations.

Supplementary Figure 5: Electron micrographs **(a)** and detail images **(b)** of *B. subtilis* cells treated with different antibiotics for 20 min.

Supplementary Figure 6: Examples of vancomycin **(a)**, nitrofurantoin **(b)**, and tetracycline-induced cell damage **(c).**

Supplementary Figure 7: Effects of 5 min treatment with nitrofurantoin on the nucleoid of *B. subtilis* 168.

Supplementary Figure 8: Localization of tetracycline and anhydrotetracycline in *B. subtilis* 168.

Supplementary Figure 9: Localization of AtpA-GFP in *B. subtilis* BS23. AtpA is a subunit of ATP synthase.

Supplementary Figure 10: Exemplary lesions observed in *B. subtilis* 168 caused by anhydrotetracycline.

Supplementary Figure 11: DiSC(3)5 staining of untreated *B. subtilis* 168 cells (negative control) and cells treated with gramicidin (1 µg/ml, positive control).

Supplementary Figure 12: DiSC(3)5 staining of *B. subtilis* 168 cells treated with tetracycline (2 µg/ml).

Supplementary Figure 13: DiSC(3)5 staining of *B. subtilis* 168 cells treated with anhydrotetracycline (2 µg/ml).

Supplementary Figure 14: Inhibition of translation does not cause membrane aberrations in *B. subtilis* 168.

Supplementary Figure 15: Inhibition of translation does not cause delocalization of the membrane potential-dependent membrane proteins MinD and MinC.

Supplementary Figure 16: Inhibition of translation does not cause delocalization of MreB.

Supplementary Figure 17: Inhibition of translation does not diminish fluid membrane domains.

Supplementary Figure 18: Effects of different tetracycline concentrations on *B. subtilis* 168 and PG112.

Supplementary Figure 19: Effect of anhydrotetracycline on tetracycline-resistant strains.

Supplementary Figure 20: DiSC(3)5 control experiments.

Supplementary References

Supplementary Table 1: Minimal inhibitory concentrations of antimicrobial compounds against *B. subtilis* 168.

compound	MIC (µg/ml)
valinomycin	16
vancomycin	0.5
ampicillin	0.5
daptomycin	1
MP196	32
nitrofurantoin	8
tetracycline	8
anhydrotetracycline	4

Supplementary Table 2: Bacterial strains used in this study.

strain name	relevant genotype	induction	reference
B. subtilis 168	-		[1]
B. subtilis MW18	*sepF::spc aprE::kan Pspac-sepF*	1 mM IPTG	[2]
B. subtilis LB318	*amyE::spc mgfp-minD aprE::cat mcherry-minC*	0.1 mM IPTG, 0.1% xylose	[3]
B. subtilis TNVS205	*aprE::cat-Pspac-mcherry-mreB*	0.3 mM IPTG	this study
B. subtilis BS23	*atpA-gfp Pxyl-'atpA cat*	0.1% xylose	[4]
B. subtilis PG112	*tet-4*	-	[5]
B. subtilis SG82	*lacA::tet*	-	[5]
E. coli MG1655	-	-	[6]
M. bovis BCG Tice	-	-	[7]
A. laidlawii PG-8A	-	-	[8]

Supplementary Table 3: Primer sequences.

name	sequence
TerS135	GGGCGTTAGCCCAAGCGCATCA
TerS337	CATGTCTGTGCAGGCTGCCGGA
TerS338	CGGCAGCCTGCACAGACATGTT
TerS397	GGCTCAGGAAGCGGCTCAGGATCCATGTTTGGAATTGGTGCTAGAGACCT
TerS398	GGATCCTGAGCCGCTTCCTGAGCCTTTGTATAATTCGTCCATTCCACCT
TerS400	ATGCGCTTGGGCTAACGCCCCCCGATTATCTAGTTTTCCCTTTGA

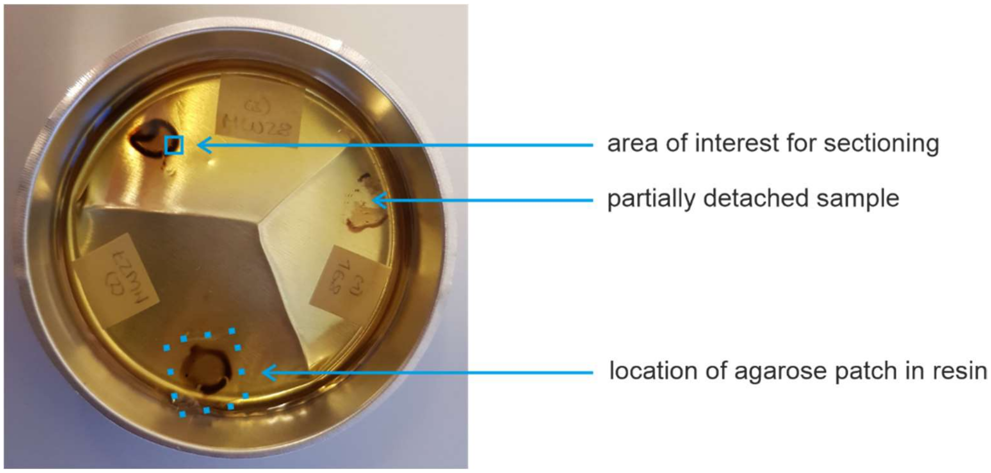

Supplementary Figure 1: Example of a polymerized EPON disc after flat embedding. The samples shown are from 150 µl logarithmically growing *B. subtilis* cultures that were pelleted and resuspended in 15 µl LB. The whole 15 µl cell suspension was spread on an agarose patch and embedded according to the single agarose layer protocol. The aluminum dish can be removed from the EPON disc and an area of interest can be cut out with a hot scalpel and mounted on a conventional EPON block for ultrathin sectioning. From these sample volumes, a minimum of 5 sectioning blocks can be prepared. Nicely aligned cells can typically be found in the middle of the sample or close to the dark halo. Within the halo itself, cells were more prone to overlap with each other, resulting in less complete longitudinally cut cells in the final sections. However, for low concentrated samples, certain mutants, and partially lysing cultures we made the experience that the dark halo gives better sections than the center of the spot. Therefore, we typically select an area of interest that contains both areas. Disc diameter 7 cm.

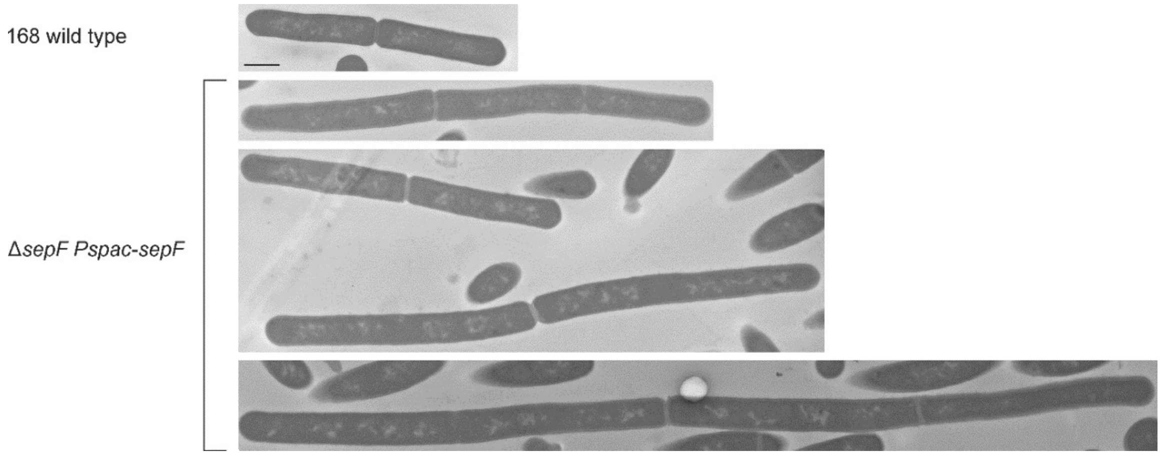

Supplementary Figure 2: Flat embedding of filamentous cells. Overexpression of the cell division protein SepF inhibits cell division by preventing septum formation[9]. Strain MW18 (*B. subtilis* 168 *sepF::spc aprE::kan Pspac-sepF*) carries an IPTG-inducible copy of the *sepF* gene in the ectopic *aprE* locus. Induction with 0.5 mM IPTG results in elongated cells[9]. Scale bar 1 µm.

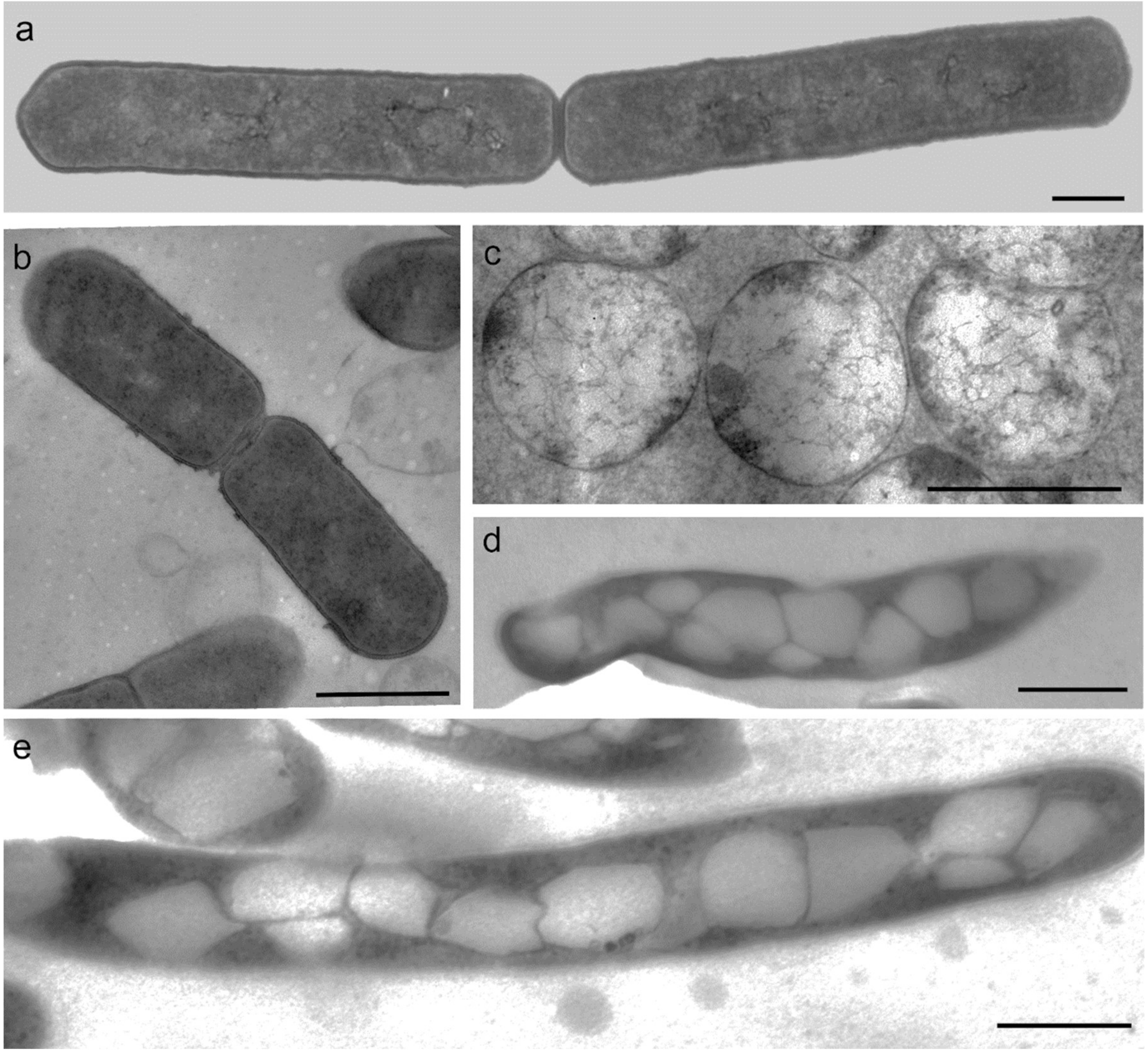

Supplementary Figure 3: Details of flat-embedded cells. **(a)** *B. subtilis*, **(b)** *E. coli*, **(c)** *A. laidlawii*, **(d)** *M. bovis* grown with 0.05% Tween 80, **(e)** *M. bovis* grown without detergent. *B. subtilis* and *M. bovis* were embedded on agarose and *E. coli* and *A. laidlawii* on glass. Scale bars 500 nm.

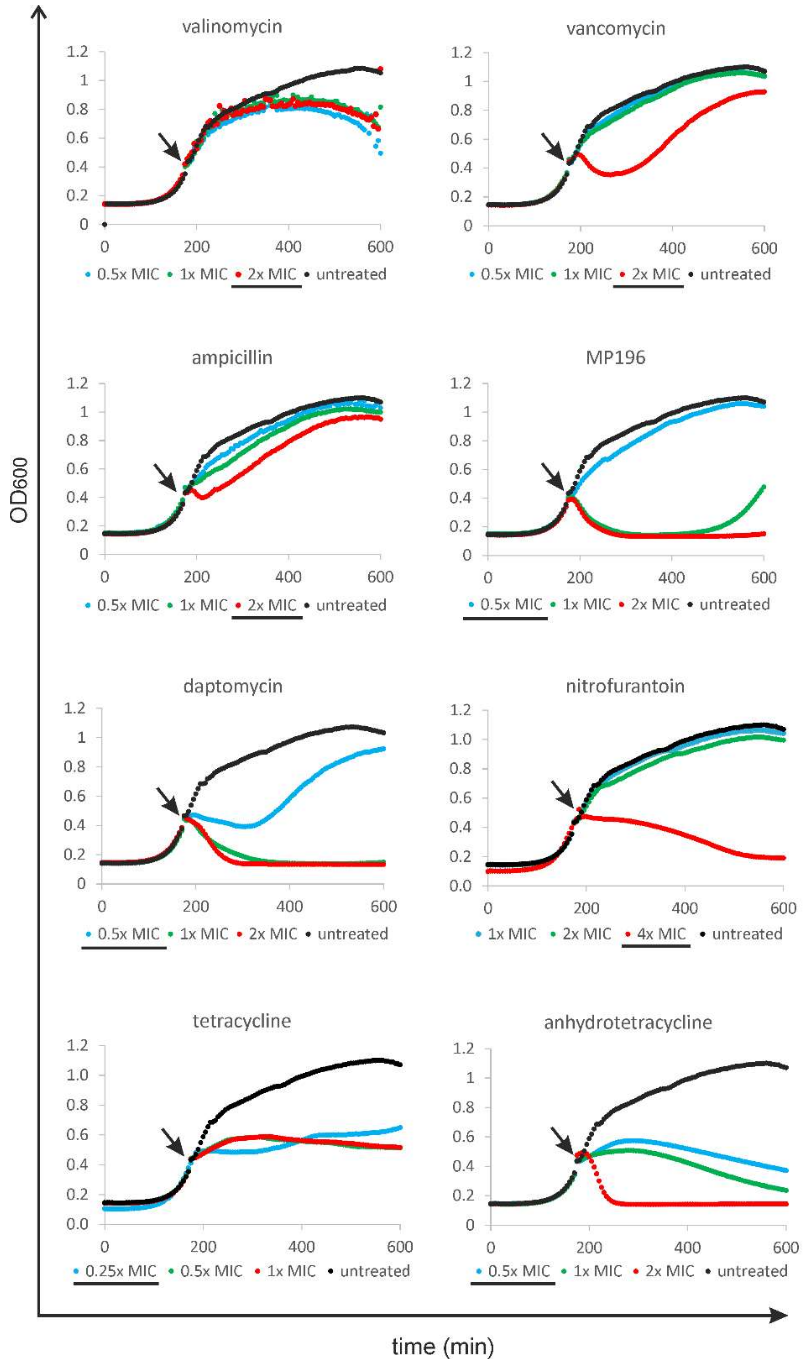

Supplementary Figure 4: Growth of *B. subtilis* 168 after treatment with different antibiotic concentrations. Arrows indicate time points of antibiotic addition. Concentrations used for further experiments are underlined. Exemplary growth curves out of three biological replicates are shown.

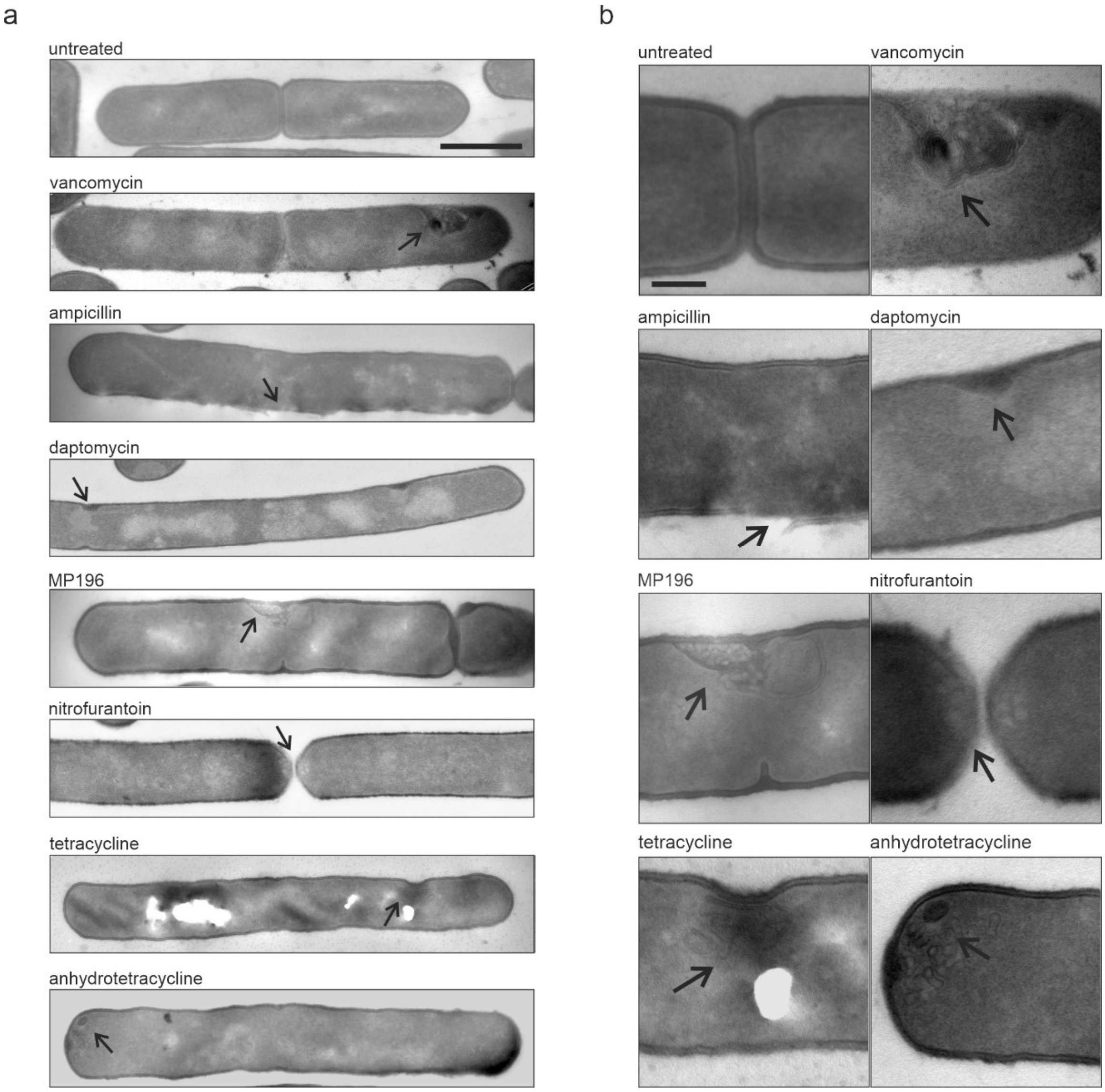

Supplementary Figure 5: Electron micrographs **(a)** and detail images **(b)** of *B. subtilis* 168 cells treated with different antibiotics for 30 min. Antibiotic-induced lesions are indicated by arrows. We mainly chose antibiotics that target the cell wall and should cause clearly visible cell wall defects. Vancomycin binds to the cell wall precursor molecule lipid II and thus inhibits cell wall synthesis. Cells treated with this antibiotic show clear cell wall lesions. Ampicillin inhibits transpeptidation of peptidoglycan polymers[10], causing cell wall thinning and ultimately cell lysis[11]. Accordingly, ampicillin-treated cells displayed partly disintegrated cell walls. Daptomycin was recently shown to hamper cell wall synthesis by targeting membrane microdomains that harbor the cell wall synthetic machinery, causing them to accumulate into lipid II-enriched foci[12,13]. In line, daptomycin-treated cells showed aberrant local cell wall thickening. The antimicrobial peptide MP196 caused intracellular cell wall structures and membrane vesicles, reflecting its dual mechanism of targeting membrane function and cell wall synthesis[14]. Nitrofurantoin is thought to kill cells by an unspecific mechanism involving oxidative damage[15]. Cells treated with this antibiotic lacked a nucleoid and showed membrane aberrations, which is consistent with oxidative damage to these cellular

structures. Tetracycline inhibits the bacterial ribosome[16]. Surprisingly, we consistently observed membrane lesions in tetracycline-treated cells. Anhydrotetracycline, an analogue of tetracycline, which is thought to rather target the cell membrane than the ribosome[17], caused similar lesions. Scale bars 1 μm (A) and 250 μm (B).

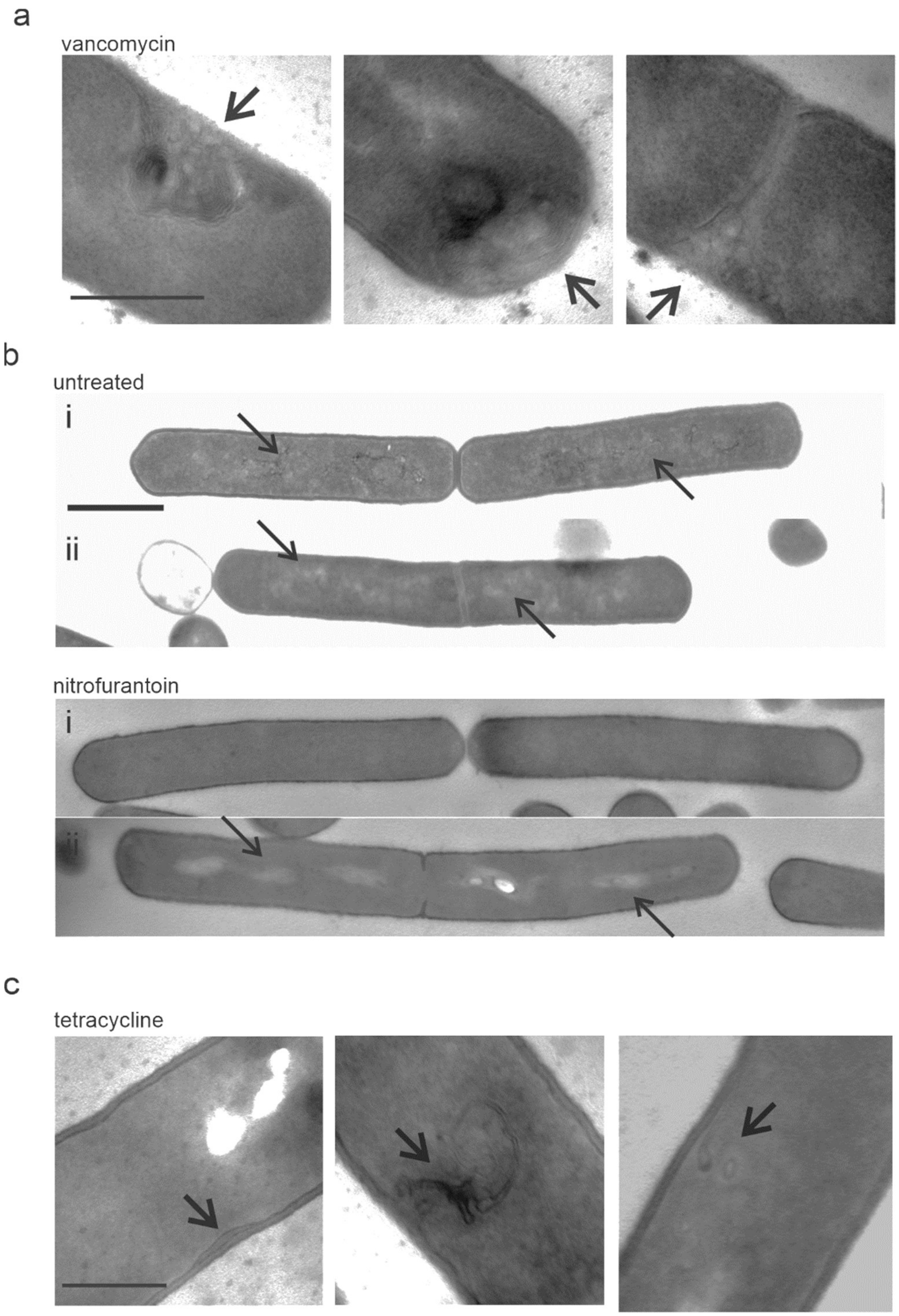

Supplementary Figure 6: Examples of vancomycin **(a)**, nitrofurantoin **(b)**, and tetracycline-induced cell damage **(c).** Scale bar 1 µm. Arrows indicate membrane lesions (a, c) or nucleoids (b).

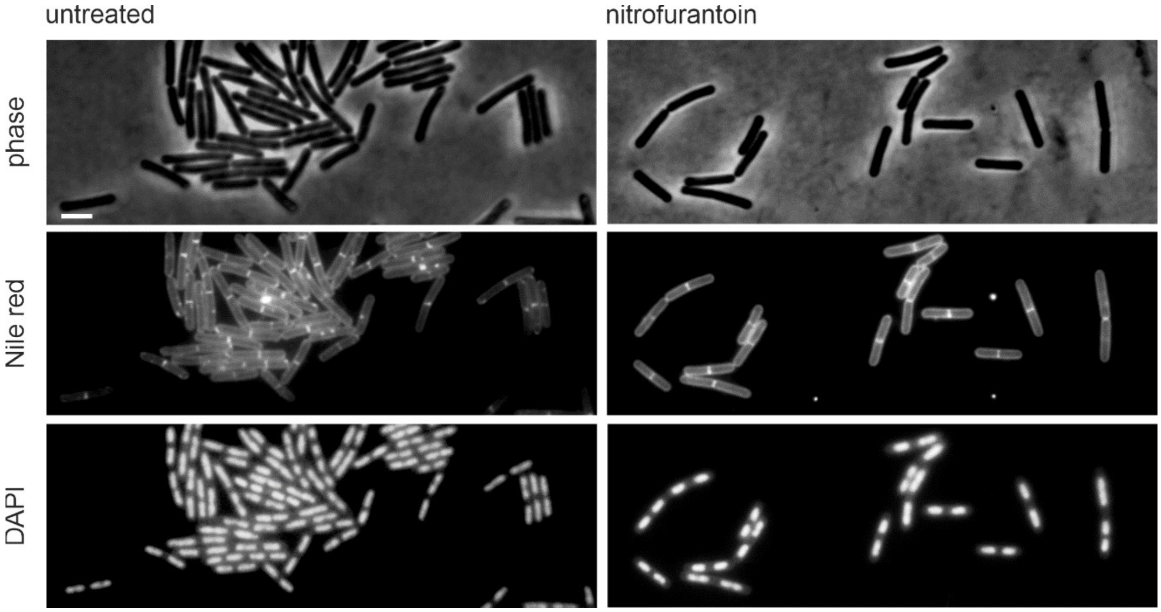

Supplementary Figure 7: Effects of 5 min treatment with nitrofurantoin on the nucleoid of *B. subtills* 168. Scale bar 2 µm.

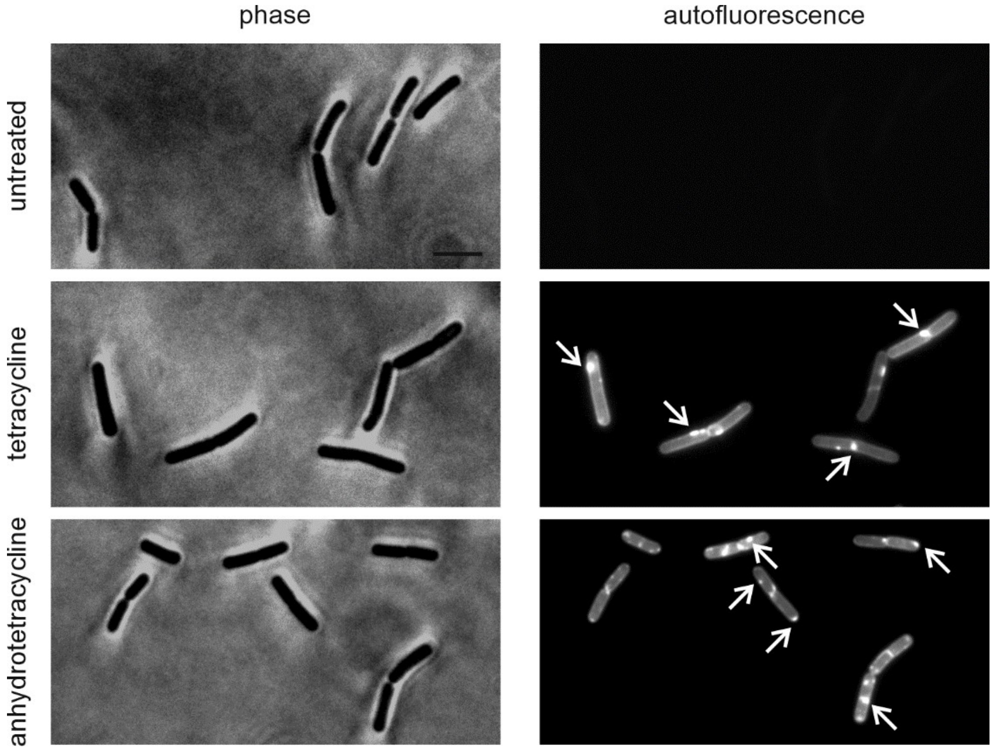

Supplementary Figure 8: Localization of tetracycline and anhydrotetracycline in *B. subtilis* 168. Green autofluorescence of the tetracyclines allows label-free localization of these antibiotics in living cells. Arrows indicate some sites of compound accumulations in the cell membrane. Scale bar 2 µm.

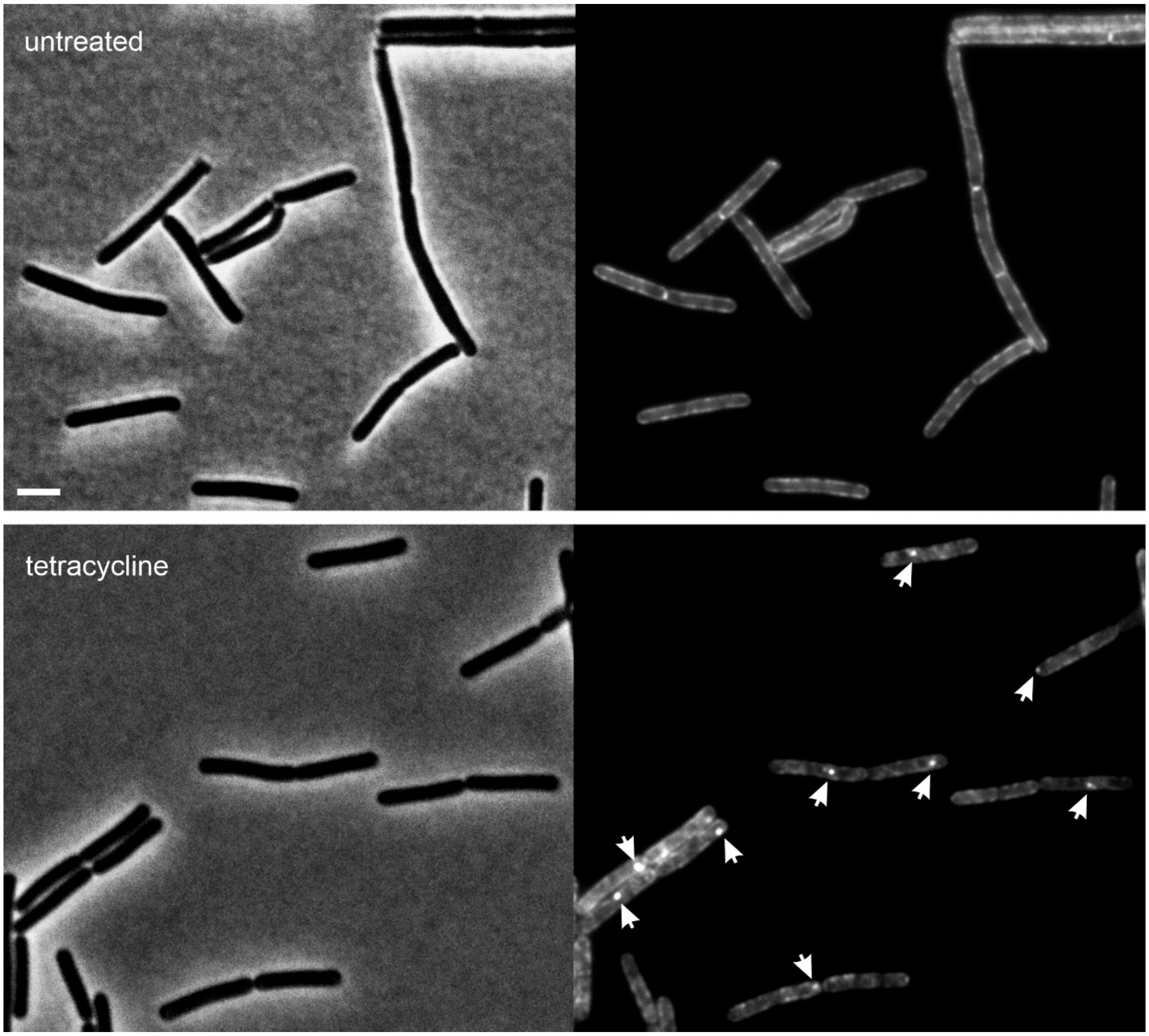

Supplementary Figure 9: Localization of AtpA-GFP in *B. subtilis* BS 23. AtpA is a subunit of ATP synthase. It is a regularly distributed membrane protein that is insensitive to disturbance of most membrane parameters but it does show an increased fluorescence signal when a double membrane is present due to invaginations[18,19]. Scale bar 2 µm.

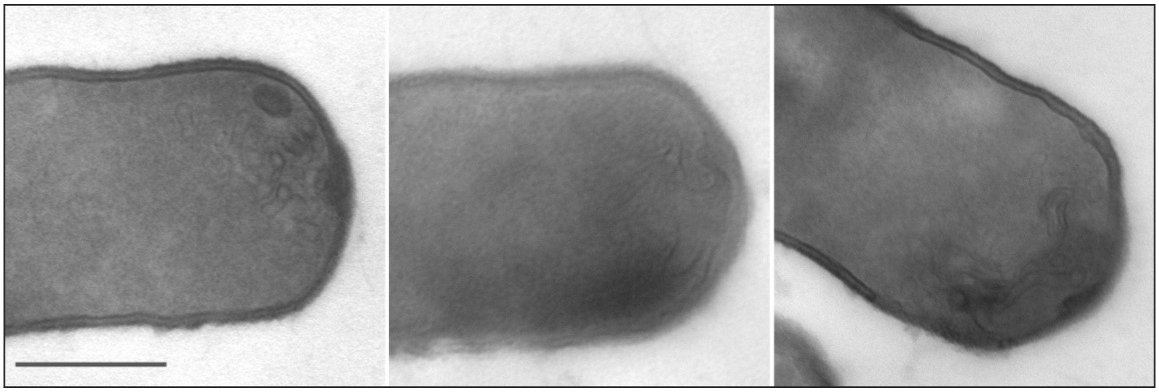

Supplementary Figure 10: Exemplary lesions observed in *B. subtilis* 168 cells caused by anhydrotetracycline. Scale bar 500 nm.

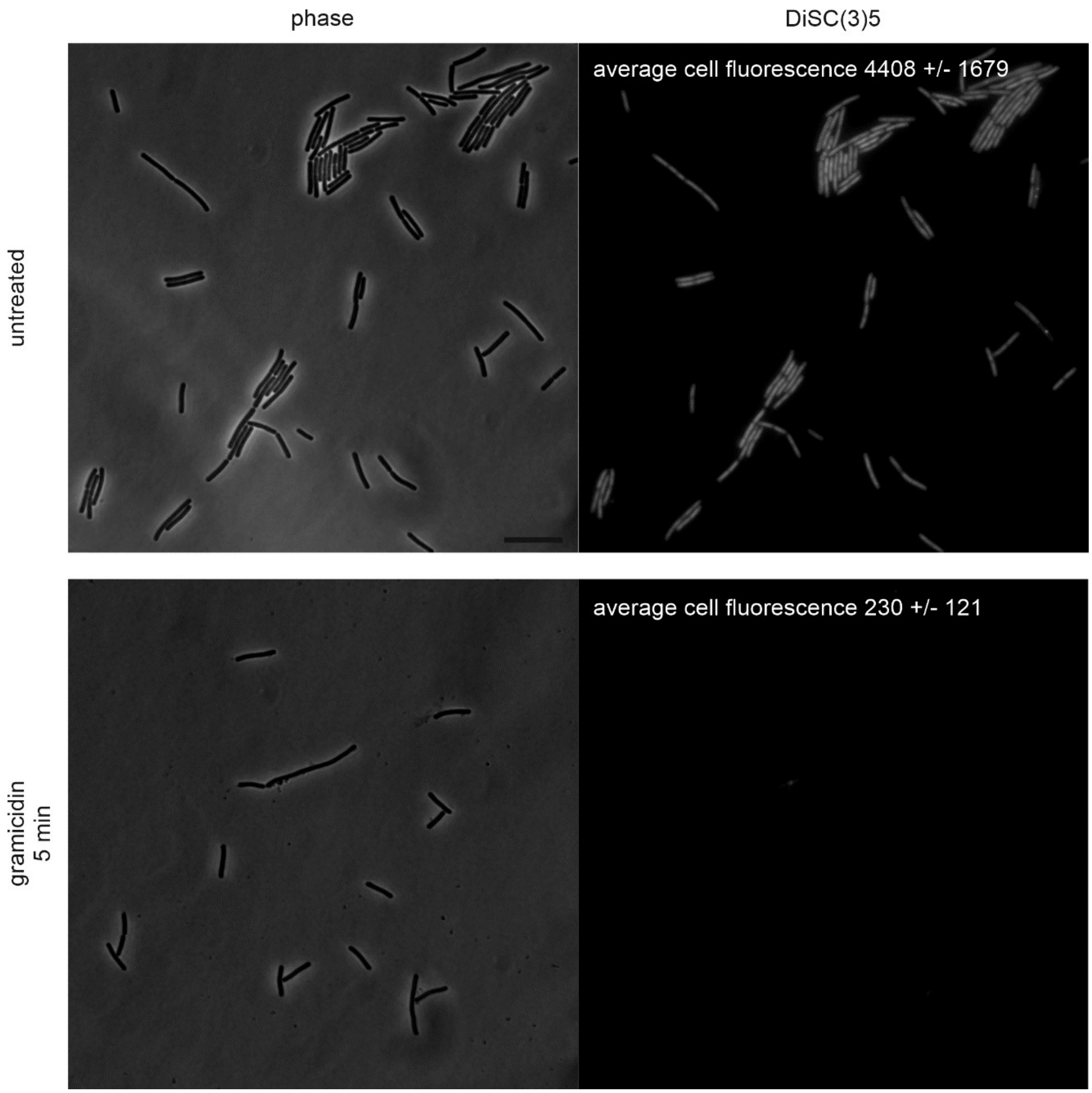

Supplementary Figure 11: DiSC(3)5 staining of untreated *B. subtilis* 168 cells (negative control) and cells treated with gramicidin (1 µg/ml, positive control). A fluorescence signal indicates the presence of a membrane potential (negative control: untreated cells). Depolarization leads to release of the dye from the cells and a diminished fluorescence signal in the cells (positive control: gramicidin). All fluorescence pictures in Supplementary Figure 11-13 have been recorded with the same exposure time and were adjusted with the same brightness and contrast settings. Average cell fluorescence was quantified from three different data sets using the ImageJ analyze particles function. Scale bar 10 µm.

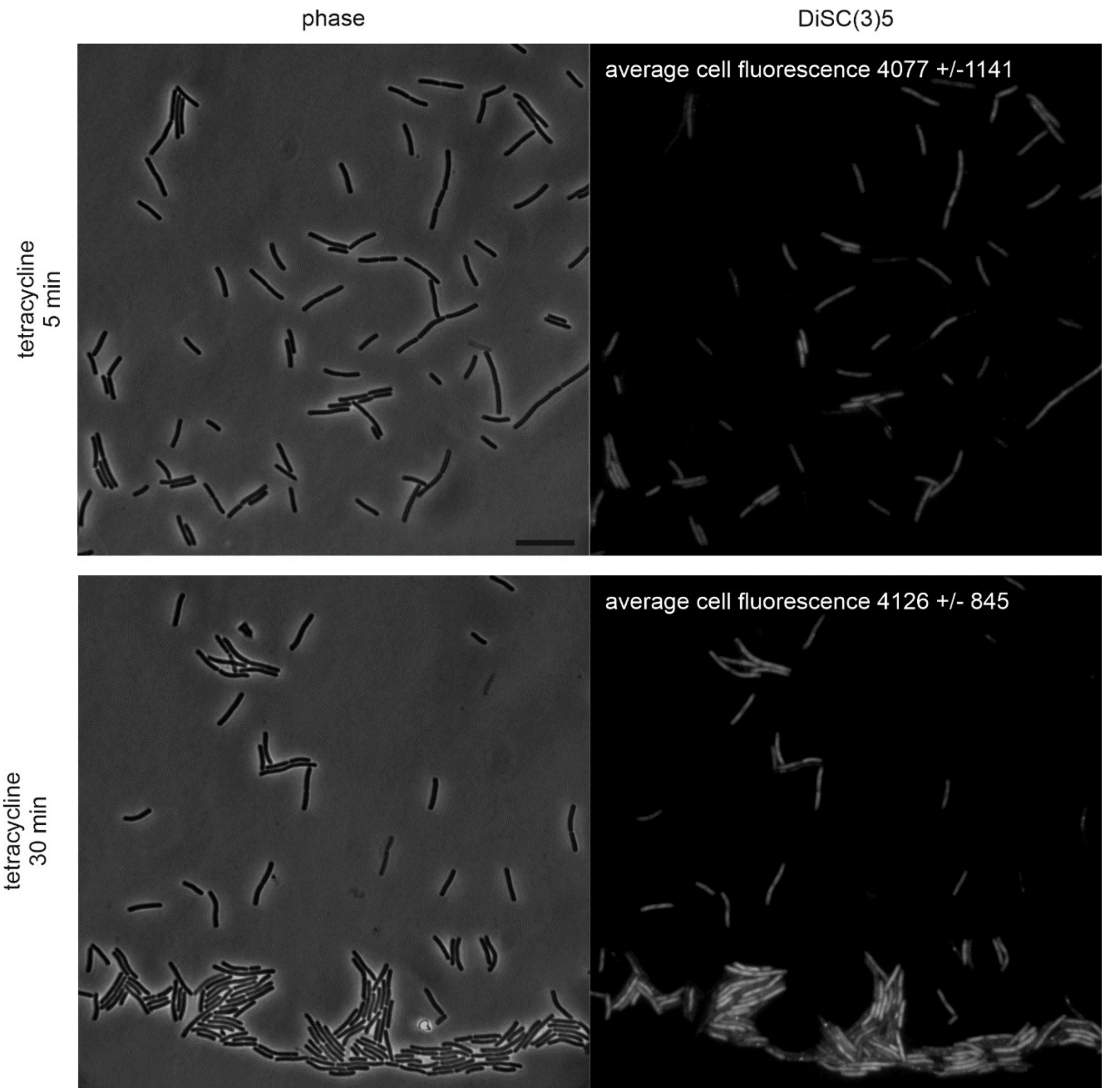

Supplementary Figure 12: DiSC(3)5 staining of *B. subtilis* 168 cells treated with tetracycline (2 µg/ml). Note the heterogeneity in the DiSC(3)5 staining. A fluorescence signal indicates the presence of a membrane potential. Depolarization leads to release of the dye from the cells and a diminished fluorescence signal in the cells. All fluorescence pictures in Supplementary Figure 11-13 have been recorded with the same exposure time and were adjusted with the same brightness and contrast settings. Average cell fluorescence was quantified from three different data sets using the ImageJ analyze particles function. Scale bar 10 µm.

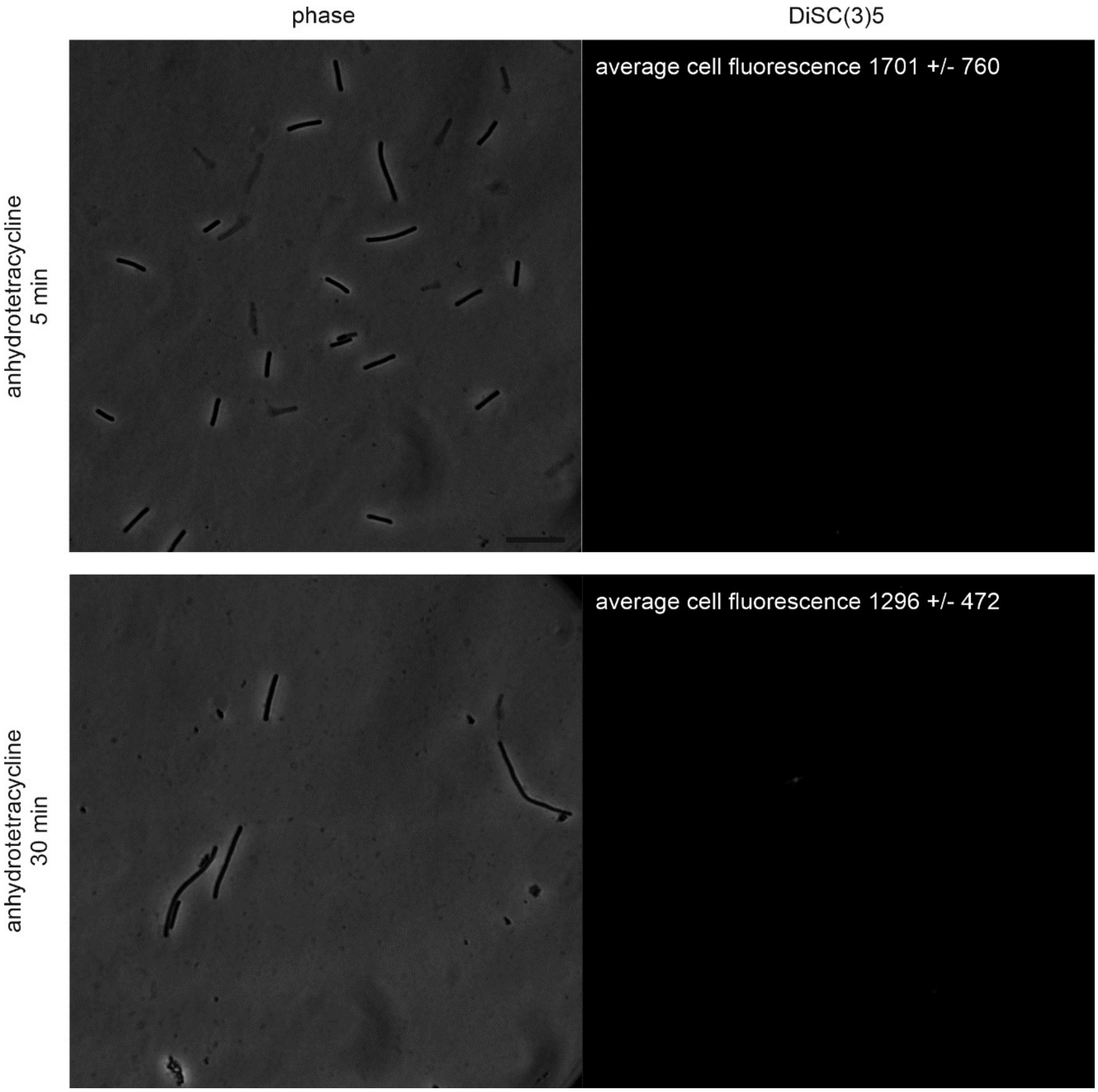

Supplementary Figure 13: DiSC(3)5 staining of *B. subtilis* 168 cells treated with anhydrotetracycline (2 µg/ml). A fluorescence signal indicates the presence of a membrane potential. Depolarization leads to release of the dye from the cells and a diminished fluorescence signal in the cells. All fluorescence pictures in Supplementary Figure 11-13 have been recorded with the same exposure time and were adjusted with the same brightness and contrast settings. Average cell fluorescence was quantified from three different data sets using the ImageJ analyze particles function. Scale bar 10 µm.

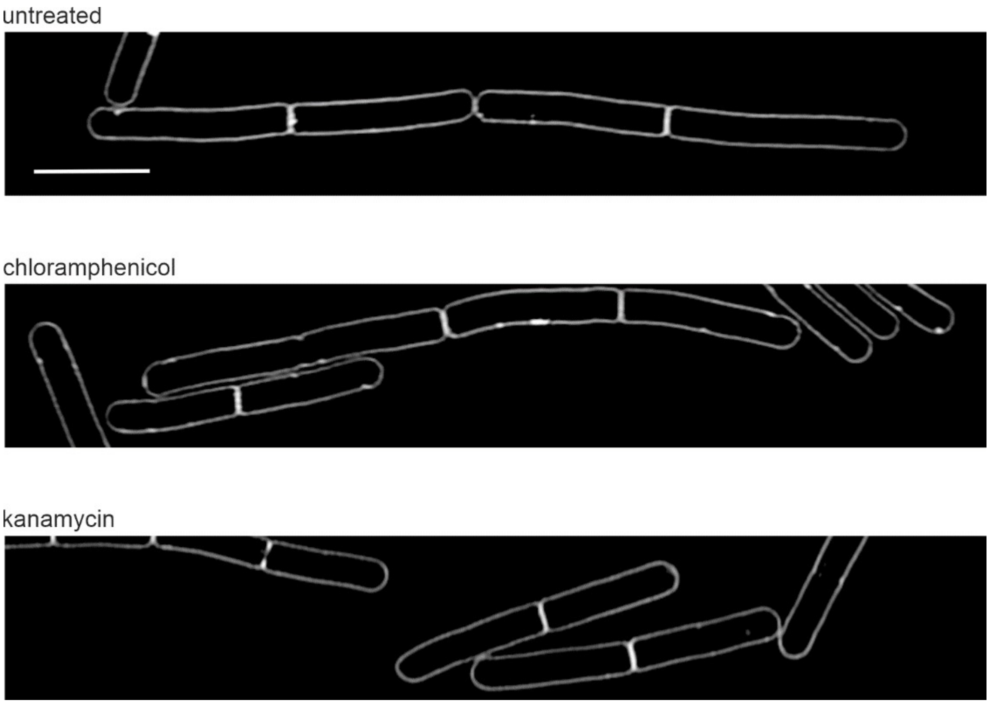

Supplementary Figure 14: Inhibition of translation does not cause membrane aberrations. Logarithmically growing *B. subtilis* 168 cultures were treated with 15 µg/ml chloramphenicol or 3 µg/ml kanamycin for 20 min, stained with Nile red, and examined by SIM microscopy. Scale bar 2 µm.

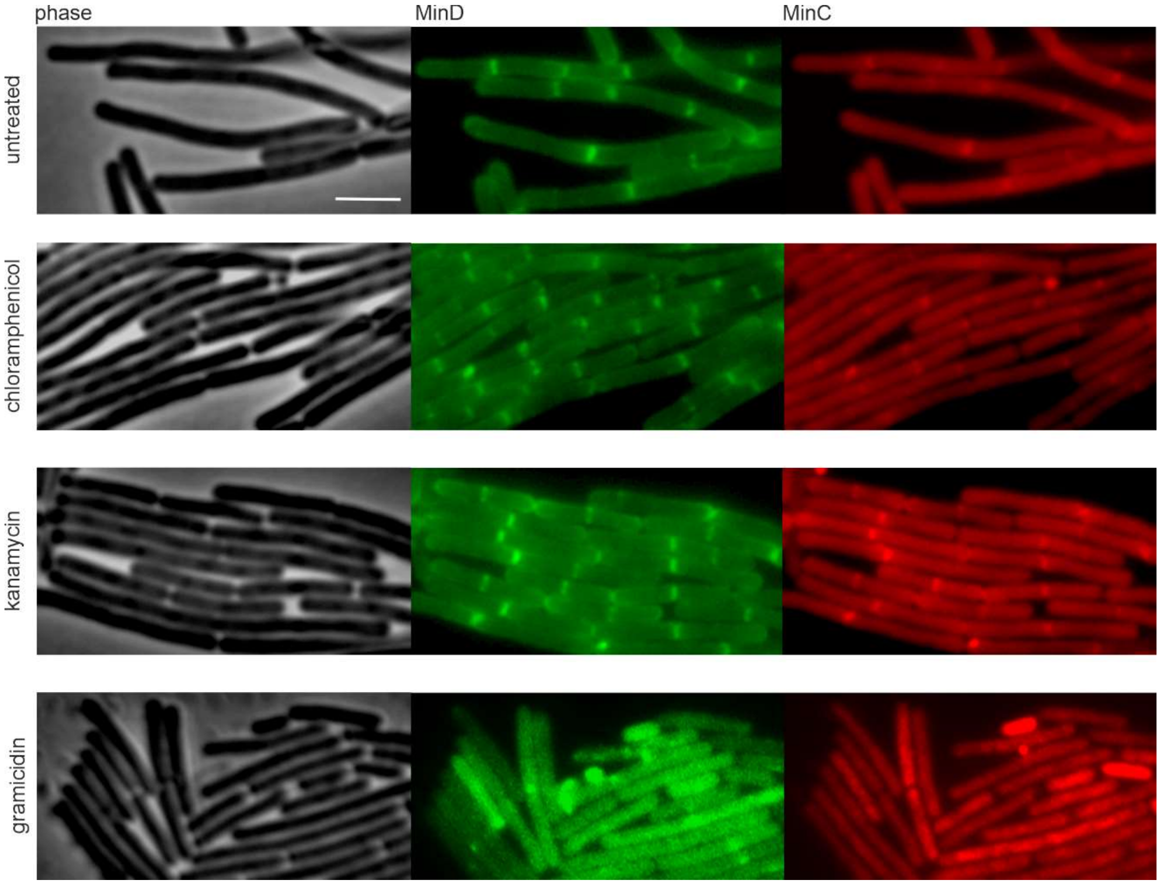

Supplementary Figure 15: Inhibition of translation does not cause delocalization of the membrane potential-dependent membrane proteins MinD and MinC. *B. subtilis* LB318, expressing GFP-MinD and mCherry-MinC, was treated with 20 µg/ml chloramphenicol, 10 µg/ml kanamycin, or 1 µg/ml gramicidin for 20 min prior to microscopy. Note that LB318 carries both a chloramphenicol and kanamycin resistance cassette. Therefore, twice the concentrations used for antibiotic selection were chosen for microscopy. Scale bar 2 µm.

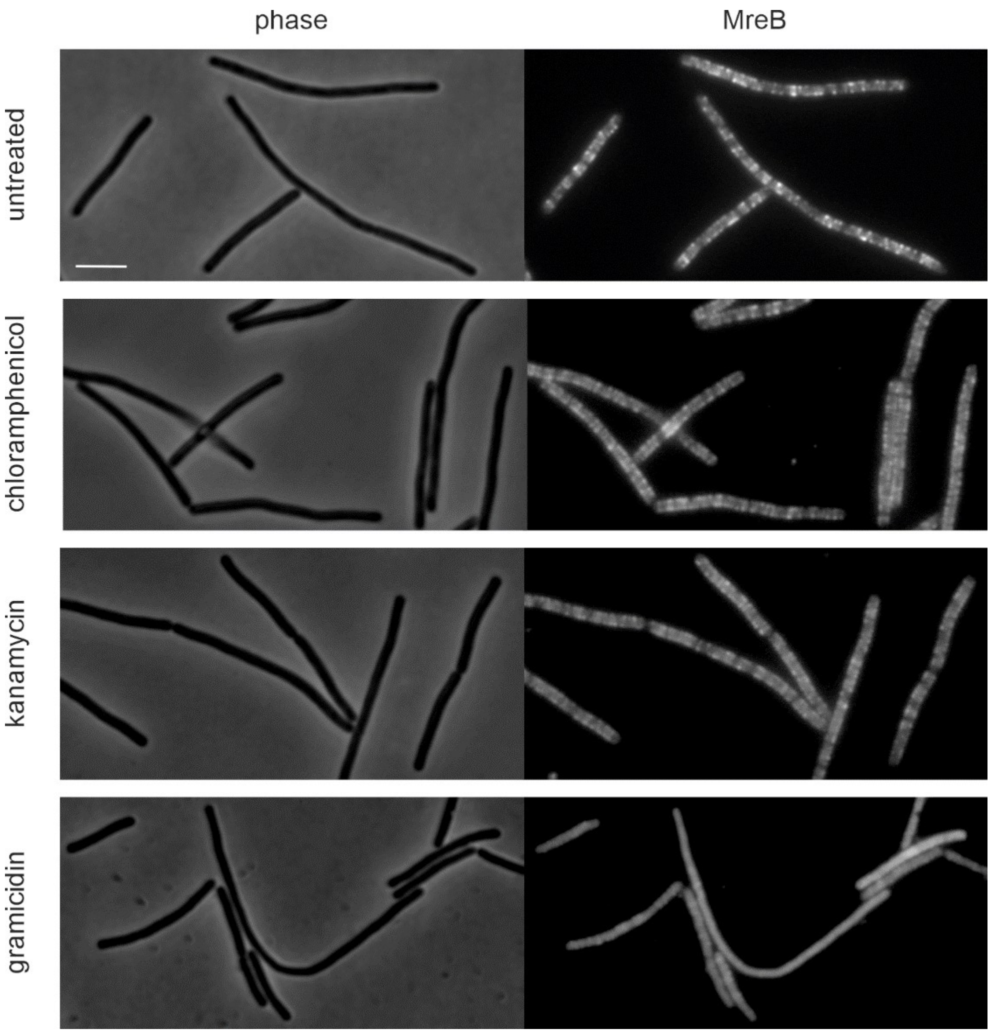

Supplementary Figure 16: Inhibition of translation does not cause delocalization of MreB. *B. subtilis* TNVS205, expressing mCherry-MreB, was treated with 20 µg/ml chloramphenicol, 3 µg/ml kanamycin, or 1 µg/ml gramicidin for 20 min prior to microscopy. Note that TNVS205 carries a chloramphenicol resistance cassette. Therefore, twice the concentration used for antibiotic selection was chosen for microscopy. Scale bar 2 µm.

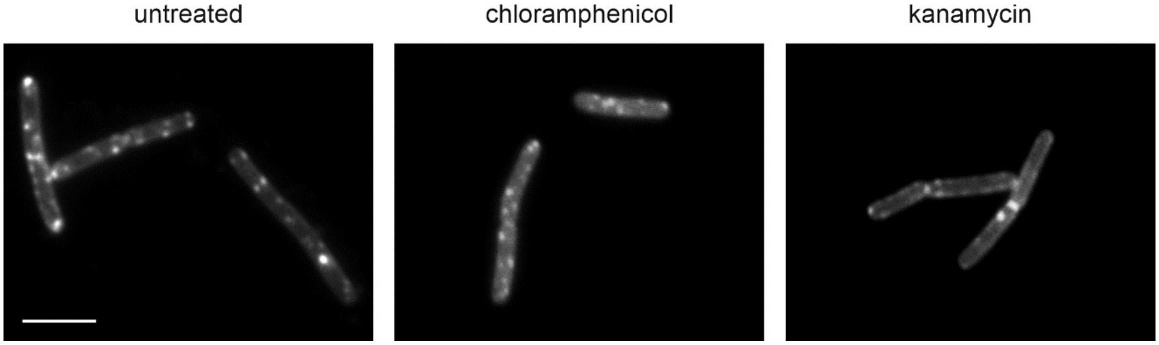

Supplementary Figure 17: Inhibition of translation does not diminish fluid membrane domains. *B. subtilis* 168 was treated with 15 µg/ml chloramphenicol or 3 µg/ml kanamycin 30 min prior to microscopy. Small effects are expected since RIFs depend on the growth phase[20] and a reduced growth rate caused by antibiotic treatment is likely to have secondary effects on RIFs. In line, RIFs were less clear after 30 min treatment with chloramphenicol and kanamycin compared to the untreated control. However, clustering or diminishing of RIFs was not observed. Scale bar 2 µm.

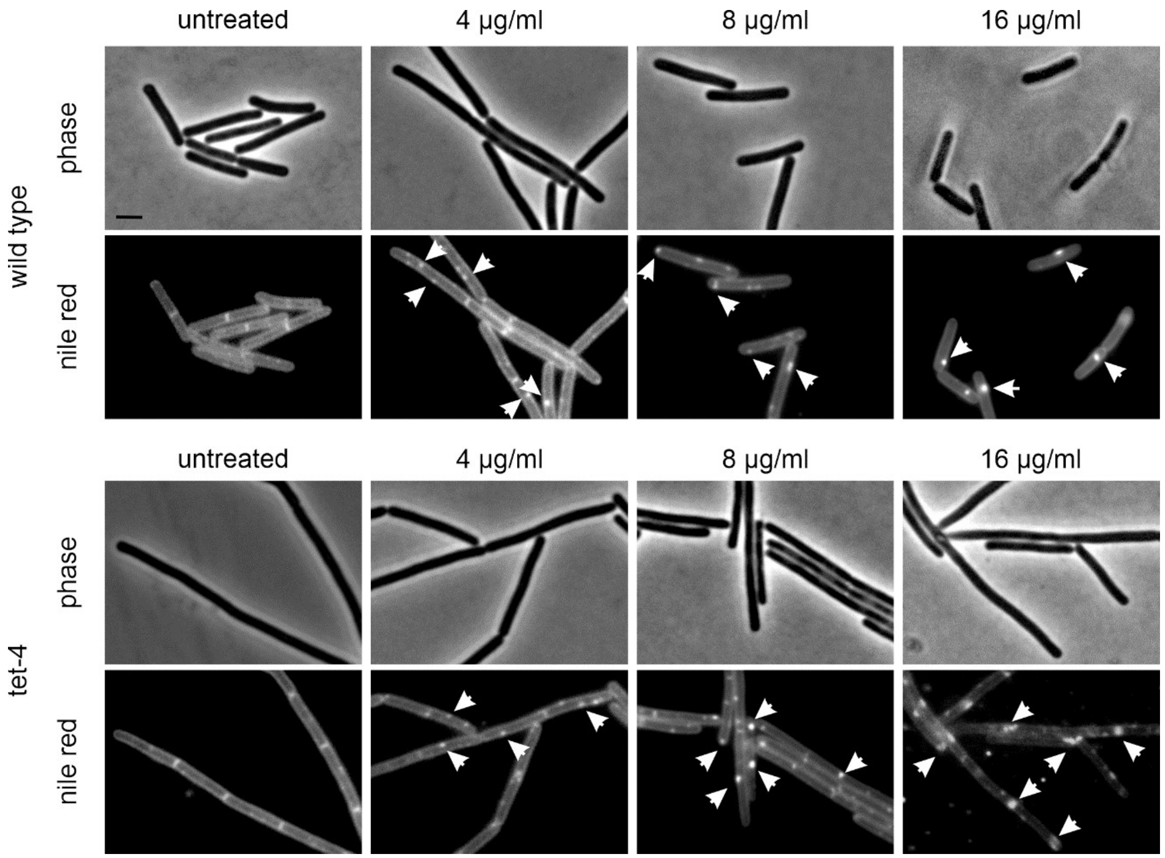

Supplementary Figure 18: Effects of different tetracycline concentrations on *B. subtilis* 168 and PG112. The tet-4 mutant strain PG112 shows the exact same phenotype as the 168 wild type at all concentrations, further corroborating the notion that the membrane activity of tetracycline is independent of ribosome inhibition. Scale bar 2 µm.

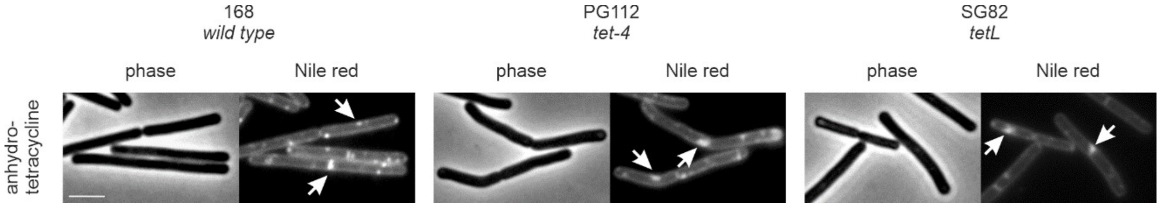

Supplementary Figure 19: Effect of anhydrotetracycline on tetracycline-resistant *B. subtilis* strains. Anhydrotetracycline is insensitive to both *tet-4* and *tetL* resistance mechanisms (MIC 1 µg/ml for both PG112 and SG82). Scale bar 2 µm.

a

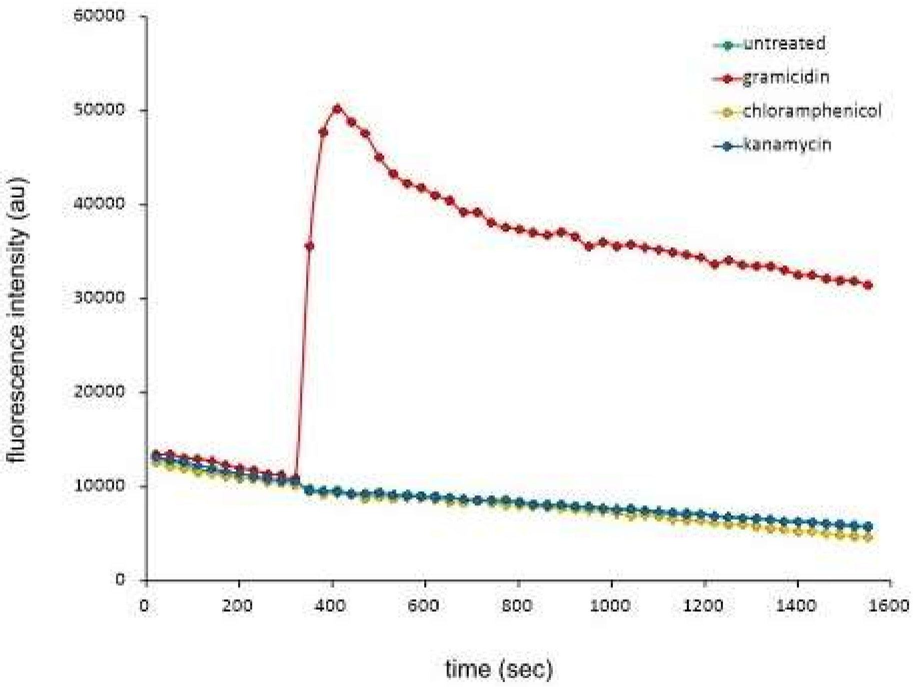

b

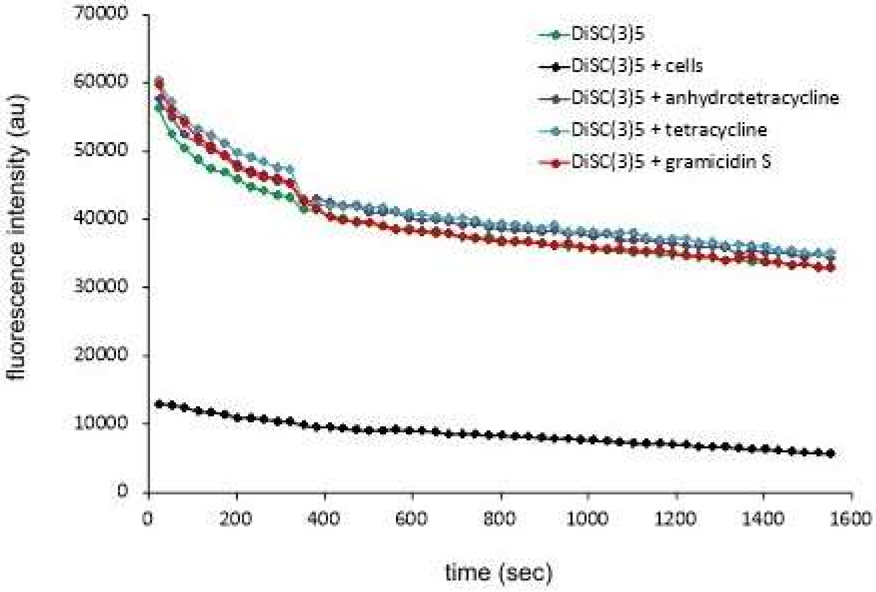

Supplementary Figure 20: DiSC(3)5 control experiments. **(a)** Inhibition of translation does not cause membrane depolarization. **(b)** Antibiotics do not affect DiSC(3)5 fluorescence in solution. DiSC(3)5 is self-quenching when accumulated in the cell membrane leading to low fluorescence in the presence of cells. In solution, fluorescence is not quenched leading to higher signal intensities. Addition of antibiotics to the dye in solution does not significantly affect its fluorescence intensity suggesting that the compounds do not interact.

Supplementary References

1. Anagnostopoulos C, Spizizen J (1960) Requirements for transformation in Bacillus subtilis. J Bacteriol 81(5):741–746.

2. Bohorquez Suarez LC (2018) The Dynamic Role of the Conserved WhiA and MinD Proteins in Chromosome Segregation, Fatty Acid Metabolism and Cell Division in Bacillus subtilis. Dissertation (University of Amsterdan). Available at: https://books.ipskampprinting.nl/thesis/524117 Bohorquez/6/.

3. Gamba P, Rietkotter E, Daniel RA, Hamoen LW (2015) Tetracycline hypersensitivity of an ezrA mutant links GalE and TseB (YpmB) to cell division. Front Microbiol 6:346.

4. Jensen KF (1993) The Escherichia coli K-12 “wild types” W3110 and MG1655 have an rph frameshift mutation that leads to pyrimidine starvation due to low pyrE expression levels. J Bacteriol 175(11):3401–3407.

5. Abdallah AM, et al. (2015) Genomic expression catalogue of a global collection of BCG vaccine strains show evidence for highly diverged metabolic and cell-wall adaptations. Sci Rep 5:15443.

6. Lazarev VN, et al. (2011) Complete genome and proteome of Acholeplasma laidlawii. J Bacteriol 193(18):4943–4953.

7. Gao Y, Wenzel M, Jonker MJ, Hamoen LW (2017) Free SepF interferes with recruitment of late cell division proteins. Sci Rep 7(1):16928.

8. Schneider T, Sahl HG (2010) An oldie but a goodie - cell wall biosynthesis as antibiotic target pathway. Int J Med Microbiol 300(2–3):161–169.

9. Schneider T, et al. (2010) Plectasin, a fungal defensin, targets the bacterial cell wall precursor Lipid II. Science 328(5982):1168–1172.

10. Mueller A, et al. (2016) Daptomycin inhibits bacterial cell envelope synthesis by interfering with fluid membrane microdomains. Proc Natl Acad Sci U S A 113:E7077-7086.

11. Pogliano J, Pogliano N, Silverman JA (2012) Daptomycin-mediated reorganization of membrane architecture causes mislocalization of essential cell division proteins. J Bacteriol 194(17):4494–4504.

12. Wenzel M, et al. (2014) Small cationic antimicrobial peptides delocalize peripheral membrane proteins. Proc Natl Acad Sci U S A 111(14):E1409-18.

13. Tu Y, McCalla DR (1975) Effect of activated nitrofurans on DNA. Biochim Biophys Acta 402(2):142–149.

14. Chopra I, Roberts M (2001) Tetracycline antibiotics: mode of action, applications, molecular biology, and epidemiology of bacterial resistance. Microbiol Mol Biol Rev 65(2):232–60 ; second page, table of contents.

15. Rasmussen B, et al. (1991) Molecular basis of tetracycline action: identification of analogs whose primary target is not the bacterial ribosome. Antimicrob Agents Chemother 35(11):2306–2311.

16. Wenzel M, et al. (2018) The multifaceted antibacterial mechanisms of the pioneering peptide antibiotics tyrocidine and gramicidin S. MBio 9(5):e00802-18.
