# Supplementary material for: A flat embedding method for transmission electron microscopy reveals an unknown mechanism of tetracycline
Source: Commun Biol. 2021 Mar 8;4:306. doi: 10.1038/s42003-021-01809-8 (PMC7940657; doi:10.1038/s42003-021-01809-8)
Supplement: Supplementary file 2 — Description of Supplementary Files [file 42003_2021_1809_MOESM2_ESM.pdf]

Description of Additional Supplementary Files

File Name: Supplementary Movie 1

Description: Preparation of agarose-embedded cells.

File Name: Supplementary Movie 2

Description: Preparation of sandwich-embedded cells.

File Name: Supplementary Data 1

Description: Source data for the main and supplementary files.
