# Supplementary material for: A flat embedding method for transmission electron microscopy reveals an unknown mechanism of tetracycline
Source: Commun Biol. 2021 Mar 8;4:306. doi: 10.1038/s42003-021-01809-8 (PMC7940657; doi:10.1038/s42003-021-01809-8)
Supplement: Supplementary file 6 — Reporting Summary [file 42003_2021_1809_MOESM6_ESM.pdf]

nature research

Corresponding author(s): Michaela Wenzel and Jan van Weering

Last updated by author(s): Dec 14, 2020

Reporting Summary

Nature Research wishes to improve the reproducibility of the work that we publish. This form provides structure for consistency and transparency in reporting. For further information on Nature Research policies, see our Editorial Policies and the Editorial Policy Checklist.

Statistics

For all statistical analyses, confirm that the following items are present in the figure legend, table legend, main text, or Methods section.

n/a	Confirmed	
☐	☒	The exact sample size (*n*) for each experimental group/condition, given as a discrete number and unit of measurement
☐	☒	A statement on whether measurements were taken from distinct samples or whether the same sample was measured repeatedly
☒	☐	The statistical test(s) used AND whether they are one- or two-sided<br>*Only common tests should be described solely by name; describe more complex techniques in the Methods section.*
☒	☐	A description of all covariates tested
☒	☐	A description of any assumptions or corrections, such as tests of normality and adjustment for multiple comparisons
☒	☐	A full description of the statistical parameters including central tendency (e.g. means) or other basic estimates (e.g. regression coefficient) AND variation (e.g. standard deviation) or associated estimates of uncertainty (e.g. confidence intervals)
☒	☐	For null hypothesis testing, the test statistic (e.g. *F*, *t*, *r*) with confidence intervals, effect sizes, degrees of freedom and *P* value noted<br>*Give P values as exact values whenever suitable.*
☒	☐	For Bayesian analysis, information on the choice of priors and Markov chain Monte Carlo settings
☒	☐	For hierarchical and complex designs, identification of the appropriate level for tests and full reporting of outcomes
☒	☐	Estimates of effect sizes (e.g. Cohen's *d*, Pearson's *r*), indicating how they were calculated

Our web collection on statistics for biologists contains articles on many of the points above.

Software and code

Policy information about availability of computer code

Data collection	All data was recorded with commercially available instrument software: iTEM for electron microscopy, NIS elements and NIS elements AR for fluorescence microscopy, and Gen5 for spectroscopy data.
Data analysis	Images were evaluated with the freely available image analasys software ImageJ. Figures were generated with commercial software: Excel for visualization of spectroscopy data and CorelDraw to arrange figures.

For manuscripts utilizing custom algorithms or software that are central to the research but not yet described in published literature, software must be made available to editors and reviewers. We strongly encourage code deposition in a community repository (e.g. GitHub). See the Nature Research guidelines for submitting code & software for further information.

Data

Policy information about availability of data

All manuscripts must include a data availability statement. This statement should provide the following information, where applicable:

- Accession codes, unique identifiers, or web links for publicly available datasets
- A list of figures that have associated raw data
- A description of any restrictions on data availability

All relevant data are contained in the manuscript and as supplementary information.

Field-specific reporting

Please select the one below that is the best fit for your research. If you are not sure, read the appropriate sections before making your selection.

☒ Life sciences ☐ Behavioural & social sciences ☐ Ecological, evolutionary & environmental sciences

For a reference copy of the document with all sections, see nature.com/documents/nr-reporting-summary-flat.pdf

Life sciences study design

All studies must disclose on these points even when the disclosure is negative.

Sample size	Electron microscopy experiments were performed in biological triplicates. Where phenotypes were quantified, a minimum of 100 cells were examined per condition per replicate. Bar charts indicate mean with standard deviation. Fluorescence microscopy experiments were performed in biological triplicates. Where phenotypes were quantified from fluorescence microscopy images, a minimum of 100 cells were examined per condition. If necessary, images from several experiments were pooled for quantification. This is indicated in the figure legends. Minimal inhibitory concentrations, growth, and spectroscopy experiments were performed in duplicates. Minimal inhibitory concentrations were identical in both replicates. Growth and spectroscopy experiments were highly similar and representative graphs are shown.
Data exclusions	For electron microscopy, only fully longitudinally cut cells were included in the analysis to ensure that phenotypes were not overlooked in diagonally or cross-sectioned cells. For fluorescence microscopy, only cells in focus were included in quantifications.
Replication	Replicate experiments yielded the same phenotypes.
Randomization	There are no experimental groups in this study that could have been randomized.
Blinding	There is no group allocation in this study that could have been blinded.

Reporting for specific materials, systems and methods

We require information from authors about some types of materials, experimental systems and methods used in many studies. Here, indicate whether each material, system or method listed is relevant to your study. If you are not sure if a list item applies to your research, read the appropriate section before selecting a response.

Materials & experimental systems

n/a	Involved in the study
☒	☐ Antibodies
☒	☐ Eukaryotic cell lines
☒	☐ Palaeontology and archaeology
☒	☐ Animals and other organisms
☒	☐ Human research participants
☒	☐ Clinical data
☒	☐ Dual use research of concern

Methods

n/a	Involved in the study
☒	☐ ChIP-seq
☒	☐ Flow cytometry
☒	☐ MRI-based neuroimaging
